# The gain and loss of long noncoding RNA associated-competing endogenous RNAs in prostate cancer

**DOI:** 10.18632/oncotarget.11128

**Published:** 2016-08-09

**Authors:** Dianming Liu, Xuexin Yu, Shuyuan Wang, Enyu Dai, Leiming Jiang, Jing Wang, Qian Yang, Feng Yang, Shunheng Zhou, Wei Jiang

**Affiliations:** ^1^ College of Bioinformatics Science and Technology, Harbin Medical University, Harbin 150081, China

**Keywords:** lncRNA, miRNA, ceRNA, prostate cancer, miRNA sponge

## Abstract

Prostate cancer (PC) is one of the most common solid tumors in men. However, the molecular mechanism of PC remains unclear. Numerous studies have demonstrated that long noncoding RNA (lncRNA) can act as microRNA (miRNA) sponge, one type of competing endogenous RNAs (ceRNAs), which offers a novel viewpoint to elucidate the mechanisms of PC. Here, we proposed an integrative systems biology approach to infer the gain and loss of ceRNAs in PC. First, we re-annotated exon microarray data to obtain lncRNA expression profiles of PC. Second, by integrating mRNA and miRNA expression, as well as miRNA targets, we constructed lncRNA-miRNA-mRNA ceRNA networks in cancer and normal samples. The lncRNAs in these two ceRNA networks tended to have a longer transcript length and cover more exons than the lncRNAs not involved in ceRNA networks. Next, we further extracted the gain and loss ceRNA networks in PC. We found that the gain ceRNAs in PC participated in cell cycle, and the loss ceRNAs in PC were associated with metabolism. We also identified potential prognostic ceRNA pairs such as MALAT1-EGR2 and MEG3-AQP3. Finally, we inferred a novel mechanism of known drugs, such as cisplatin, for the treatment of PC through gain and loss ceRNA networks. The potential drugs such as 1,2,6-tri-O-galloyl-beta-D-glucopyranose (TGGP) could modulate lncRNA-mRNA competing relationships, which may uncover new strategy for treating PC. In summary, we systematically investigated the gain and loss of ceRNAs in PC, which may prove useful for identifying potential biomarkers and therapeutics for PC.

## INTRODUCTION

Prostate cancer (PC) is the second most frequently diagnosed cancer and the six leading cause of cancer death in males worldwide [1]. Although a decrease in the death rate of patients with PC largely reflects improvements in early detection and treatment [2, 3], its etiology remains obscure.

In recent years, more and more studies have turned their attention to non-coding RNAs (ncRNAs). MicroRNAs (miRNAs) are small non-coding RNA molecules ~ 22 nucleotides in length, which are involved in RNA silencing and post-transcriptional regulation of gene expression [4]. MiRNAs play important roles in multiple biological processes, including cell development, metabolism, proliferation, differentiation and apoptosis [5, 6], and their expression has been associated with many diseases and can be altered by environmental factors, kinase and small molecule inhibitors [7, 8]. Many studies have found that mRNA can reduce miRNA bioavailability by inhibiting targeted mRNA expression, and acting as competing endogenous RNAs (ceRNAs) [9]. For example, PTEN could crosstalk with other RNAs by competing for binding to the shared miRNAs [10]. Recently, long non-coding RNAs (lncRNAs), endogenous transcribed RNA molecules longer than 200 nucleotides in length and lacking protein-coding capacity, have been a hotspot in biomedical research [11]. LncRNAs play important roles in tumorigenesis and progression [12]. Wang *et al*. have found that the lncRNA LOC400891 promoted tumor progression and was associated with a poor prognosis in PC [13]. Moreover, lncRNA can also act as ceRNA for competing miRNA [14]. Currently, increasing evidence has been provided that lncRNAs as ceRNAs are involved in disease onset and progression. For example, lncRNA H19-ZEB1 ceRNA pair has been demonstrated to regulate cell proliferation and migration in gastric cancer [15] and Yu *et al* also have found that GAS5 acted as a ceRNA of miR-222 can increase p27 expression level, and thus inhibit liver fibrosis progression [16]. Although previous reports have focused on the identification of lncRNAs in PC, the study of lncRNA as ceRNA in PC is still in its infancy.

In this study, we proposed an integrative systems biology approach to investigate the gain and loss of ceRNAs in PC. By analyzing the gain and loss ceRNA networks, we identified the survival-associated ceRNAs, which may be novel prognostic markers. Furthermore, we also found some drugs that targeted the miRNAs and influenced the ceRNAs, which may be candidate therapeutics for the treatment of PC.

## RESULTS

### Cancer and normal ceRNA networks

**Figure 1:**
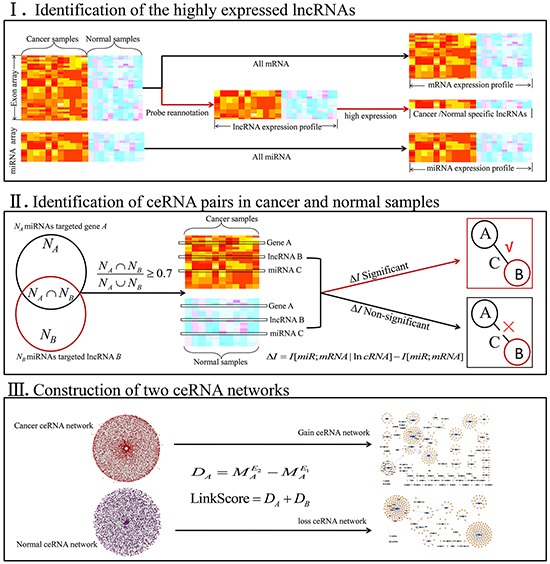
Work flow to construct ceRNA networks The process involved three steps. First, we identified the highly expressed lncRNAs. Second, ceRNA pairs were identified in cancer and normal samples. Third, we assembled all significant ceRNA pairs to construct ceRNA networks.

We proposed a pipeline to gradually identify significant lncRNA-miRNA-mRNA triples and assembled these triples into a ceRNA network, where nodes represented lncRNAs/mRNAs and edged represented their ceRNA relationships (Figure 1). We applied this approach to the PC dataset. Based on the probe reannotation, we obtained lncRNA expression data from exon microarray. Overall, we obtained 4077 lncRNAs, 17,009 mRNAs and 374 miRNAs from GSE21032 dataset. A previous study had demonstrated that highly expressed lncRNAs more likely acted as miRNA sponges [17]. Thus, we selected the top 200 (top 5%) highly expressed lncRNAs in PC and normal samples (Supplementary Table S1). There were 116 miRNAs that satisfied the criteria (see Methods) of interaction with these highly expressed lncRNAs. We then used the difference of mutual information and conditional mutual information (CMI) Δ*I* to evaluate whether one lncRNA in certain triple acted as miRNA sponge. Moreover, permutation test was used to calculate the significance level for each triple [18]. The triple with the significance level of *P* value < 0.01 was used for constructing ceRNA network. At last, there were 13062 triples in cancer ceRNA network and 9374 triples in normal ceRNA network (Supplementary Figure S1).

### Properties of lncRNAs in ceRNA networks

**Figure 2:**
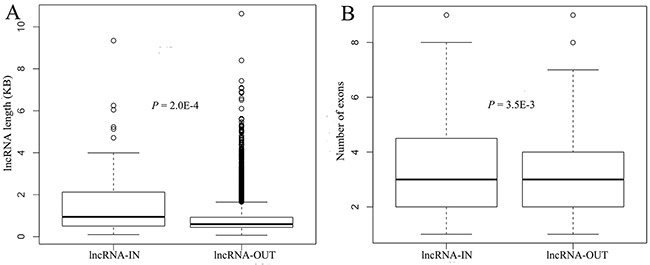
The properties of lncRNAs in ceRNA networks **A.** the boxplot depicted the length of lncRNAs in the ceRNA networks compared with lncRNAs not involved in the ceRNA networks. **B.** the boxplot depicted the number of lncRNA exons in the ceRNA networks compared with lncRNAs not involved in the ceRNA networks. P values were determined by the Mann-Whitney U test. The ‘lncRNA-IN’ represented lncRNAs in the ceRNA networks, and the ‘lncRNA-OUT’ represented lncRNAs that were not in the ceRNA networks.

We explored the transcript length and exon number of lncRNAs in the ceRNA networks (lncRNA-IN), and compared these properties with those of lncRNAs not involved in the two ceRNA networks (lncRNA-OUT). Transcripts for lncRNA-IN were 1.8-fold longer than lncRNA-OUT (average lengths: 1683 nt for lncRNA-IN versus 935 nt for lncRNA-OUT; *P* value= 2.0×10^-4^; Figure 2A). Moreover, lncRNA-IN had more exons per transcript than lncRNA-OUT (4 versus 3; *P* value= 3.5×10^-3^; Figure 2B). Wang *et al*. found similar characteristics for lncRNAs in their study [19]. These results suggested that long transcript and a larger numbers of exon may be involved in the function as miRNA sponges in biological processes.

### Gain and loss of ceRNA in PC

**Figure 3:**
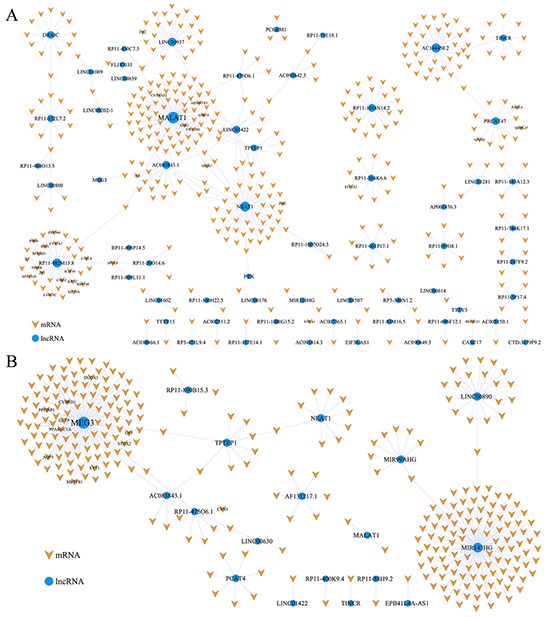
The gain and loss ceRNAs network **A.** the gain ceRNAs network; **B.** the loss ceRNAs network. The orange nodes indicated mRNAs, and the blue nodes indicated lncRNAs. Some representative nodes were named.

To determine the gain and loss ceRNA networks, we used a Cytoscape plug-in ‘ExprEssence’ to further filter the two ceRNA networks and identify which ceRNA associations were gained in the cancer network or lost in the cancer network leading to the disease (see Methods). The cancer ceRNA network filtered as gain ceRNA network contained 383 mRNAs, 100 miRNAs and 62 lncRNAs (Figure 3A, Supplementary Table S2). The normal ceRNA network filtered as loss ceRNA network contained 298 mRNAs, 84 miRNAs and 18 lncRNAs (Figure 3B, Supplementary Table S3). The ceRNA association gained or lost in the ceRNA network may predispose individuals to develop cancer.

**Figure 4:**
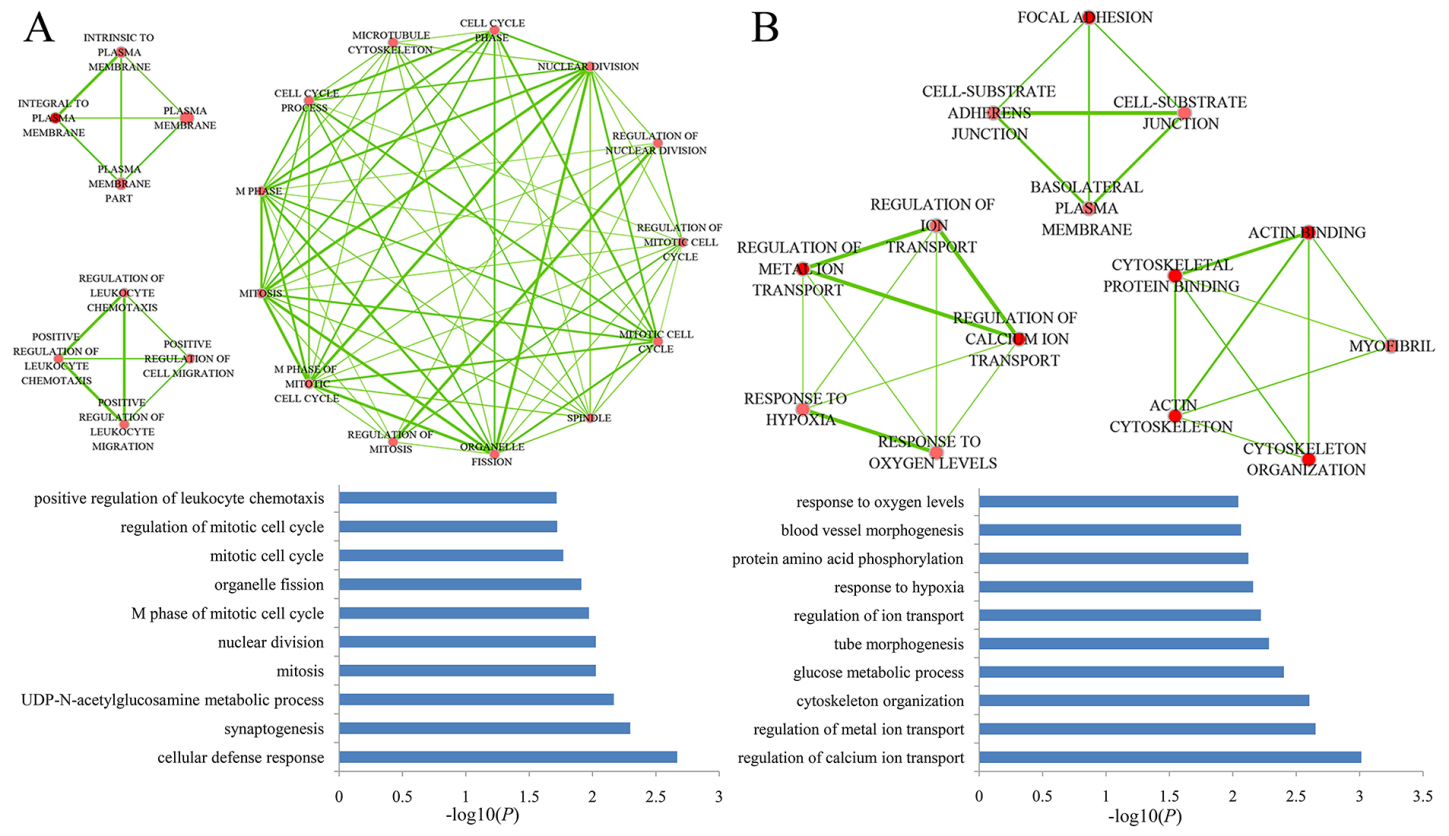
Significantly enriched GO terms in the gain and loss ceRNA networks **A.** significantly enriched GO terms in the gain ceRNA network. **B.** significantly enriched GO terms in the loss ceRNA network. The nodes of two networks indicated GO terms, and the edges indicated two GO terms with shared genes.

Gene ontology (GO) functional enrichment analysis was performed to identify the significantly enriched biological processes in the gain and loss ceRNA networks using DAVID [20]. Because the functions of most lncRNAs were poorly defined, we only conducted the functional annotation analysis of mRNAs. The significantly enriched GO terms (*P*<0.05) were shown in Figure 4A. In the gain ceRNA network, the functions of these genes mostly consisted of mitosis and mitotic cell cycle as these processes were necessary to PC cell proliferation [21, 22]. Interestingly, we also found that positive regulation of cell migration was closely associated with PC metastasis [23]. Thus, the function of the gain ceRNA network played a crucial role in PC. For the loss ceRNA network, these genes were involved in glucose metabolism, response to oxygen levels and the regulation of ion transport (Figure 4B), all functions associated with metabolic processes [24-26]. Furthermore, we found that 12 PC-specific tumor suppressor genes and lncRNAs in the loss ceRNA network (Supplementary Table S4), and the proportion of PC-specific tumor suppressor genes in the loss ceRNA network was significantly higher than all PC-specific tumor suppressor genes in all human genes (hypergeometric *P* value = 0.0177). This result suggested that the loss ceRNA network might play a key role in suppressing the occurrence and development of PC.

### Prognostic ceRNAs in PC

**Figure 5:**
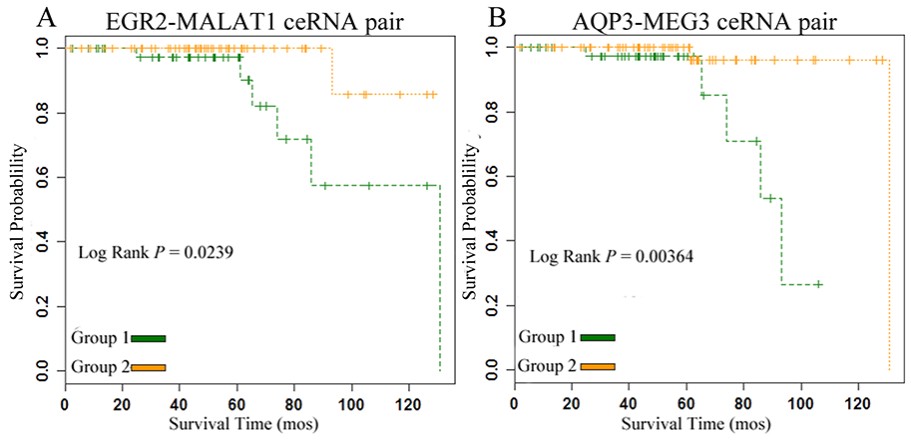
ceRNA pairs were significantly correlated with the overall survival of PC patients in the gain and loss ceRNA networks **A.** gain ceRNA pair. **B.** loss ceRNA pair.

For each biological network, a crucial characteristic was its connectivity, which reflected how often a node interacted with other nodes. Hub nodes whose connectivity was extremely high were always very crucial nodes [27]. In the ceRNA network, we defined the nodes with a degree of connectivity greater than 15 as hub nodes. Thus, we sorted the connectivity of each node in the gain ceRNA network to identify important nodes. The lncRNA metastasis associated lung adenocarcinoma transcript 1 (MALAT1) was the hub node in the gain network. MALAT1 was a known prostate cancer gene [28]. We found that MALAT1 acted as ceRNA in the gain ceRNA network, which regulated the expression of 104 mRNAs. Interestingly, MALAT1 acted as miRNA sponge to adsorb miRNAs and weakened the inhibition of early growth response 2 (EGR2) expression. They were bound by miR-93, a known prostate cancer-related miRNA [29]. We also found that the expression of MALAT1-EGR2 ceRNA pair significantly correlated with the overall survival of patients (Figure 5A). Therefore, we suspected that the MALAT1-EGR2 ceRNA pair could be a candidate therapeutic target of PC. Moreover, the mRNA G protein-coupled receptor 19 (GPR19) strongly expressed in PC. We found that lncRNA MALAT1 also acted as miRNA sponge to weaken the inhibition of GPR19 expression, they were bound by miR-30d, and the miRNA was a prostate cancer-related miRNA [30]. The expression of MALAT1-GPR19 pair significantly correlated with the overall survival of PC patients (Supplementary Figure S2). All of the prognostic ceRNA pairs are summarized in Supplementary Table S5.

Furthermore, the lncRNA MEG3 interacted with 157 neighbors: this node had the largest degree of connectivity in the loss ceRNA network, interacting with 115 different mRNAs and 50 different miRNAs. Recently, many studies had found that maternally expressed 3 (MEG3) inhibited non-small cell lung cancer (NSCLC) cell proliferation and that MEG3 was associated with a poor prognosis and promoted cell proliferation in gastric cancer [31, 32]. MEG3 was down-regulated in many diseases, such as NSCLC, gastric cancer and bladder cancer [33]. However, little was known of the lncRNA of PC. In our study, MEG3 was an important hub node in the loss ceRNA network. We extracted all interactions between MEG3 and mRNAs, and found that the MEG3-mRNA ceRNA pairs had prognostic value. For example, MEG3-AQP3 was a ceRNA pair that could significantly distinguish the cancer samples with different overall survival times (Figure 5B). MEG3 acted as the miRNA sponge to adsorb miRNA and weaken the inhibition of AQP3 expression. AQP3 had been found to facilitate the transport of nonionic small solutes such as urea and glycerol, and the expression level of AQP3 in prostate was significantly lower in the diabetic model groups [34]. All prognostic ceRNA pairs in the loss network were shown in Supplementary Table S6. These results suggested that the structure analysis of the gain and loss ceRNA networks was an efficient method for detecting prognostic biomarkers for PC.

### Potential small molecule drugs for PC treatment

**Figure 6:**
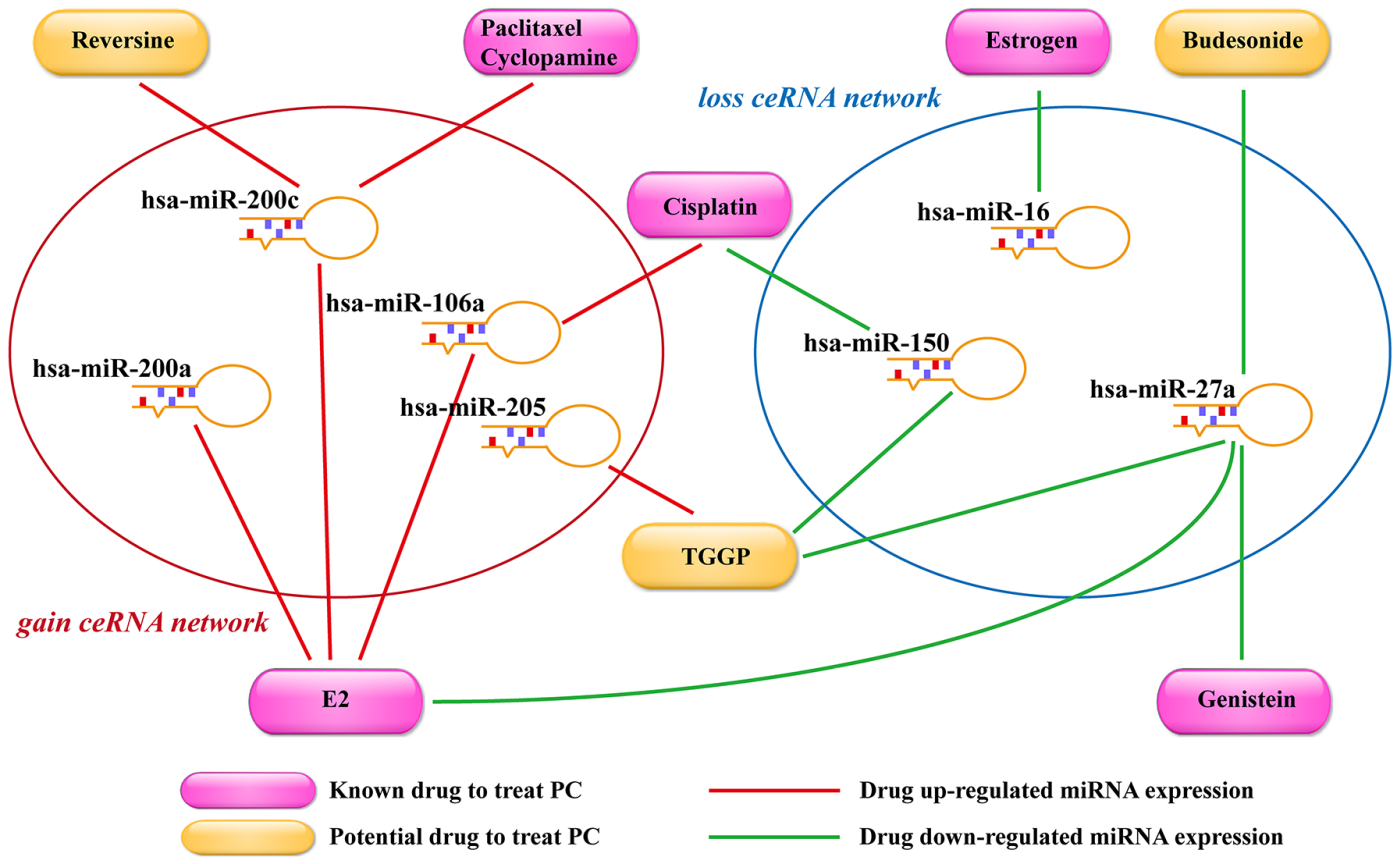
Potential small molecule drugs for PC treatment The orange nodes indicated potential PC treatment drugs, and the red nodes indicated FDA-approved drugs for PC treatment. Red lines represented the drug up-regulated the expression of miRNAs, and the green lines represented the drug down-regulated the expression of miRNAs.

In the gain and loss ceRNA networks, the perturbation of miRNA expression could influence the expression level of many lncRNAs and mRNAs. The gain and loss ceRNA networks significantly contained PC-related miRNAs (*P* values were smaller than 2.2×10^-16^) verified by HMDD [35]. Moreover, previous studies had found that bioactive small molecules could regulate miRNA expression. For example, SM2miR provided a fairly comprehensive repository of the influences of small molecules on miRNA expression [36]. Thus, based on the gain and loss ceRNA networks and the information in SM2miR, we inferred that some potential drugs could be used for the treatment of PC patients. In the gain ceRNA network, these potential drugs could up-regulate the miRNA expression and further down-regulate the expression of the corresponding mRNA/lncRNA and help in the treatment of PC, such as paclitaxel cyclopamine (up-regulated miR-200c), 17beta-estradiol (E2) (up-regulated miR-106a/200a/200c) and TGGP (up-regulated miR-205; Figure 6 and Supplementary Table S7). Recent studies had demonstrated that paclitaxel cyclopamine and E2 had been used for the treatment of PC: they not only slowed down tumor growth but also significantly improved the survival time in PC [37, 38]. In addition, TGGP inhibited cell proliferation and increased apoptosis in liver cancer cells [39]. Thus, we inferred that TGGP might be a novel potential drug for the treatment of PC. In the loss ceRNA network, these potential drugs could down-regulate the miRNA expression and up-regulate the expression of the corresponding mRNA/lncRNA for the treatment of PC, such as cisplatin (down-regulated miR-150), estrogen (down-regulated miR-16) and budesonide (down-regulated miR-27a). Cisplatin is a chemotherapy drug used for treating various types of solid tumors, such as prostate cancer, ovarian cancer and bladder cancer [40-42]. In this study, we identified the GSMT2-MEG3 ceRNA pair based upon competing miR-150. A previous study had demonstrated that cisplatin could up-regulate the expression of GSTM2, but the detail mechanism was uncertain. Based on the loss ceRNA network, we hypothesized that cisplatin dysregulated the miR-150 expression and further perturbed the expression of ceRNA (GSMT2 and MEG3). Furthermore, Luo *et al*. has shown that MEG3 can inhibit cell proliferation and induce apoptosis in PC [43].

## DISCUSSION

In this study, we proposed a novel computational approach suitable to explore the potential role of lncRNAs as miRNA sponges for preserving homeostasis and preventing disease. We applied our method to identify two ceRNA networks in cancer and normal samples. We then removed the common lncRNA-mRNA pairs that appeared in both ceRNA networks and determined the gain and loss ceRNA networks. Furthermore, we found that some lncRNAs acting as miRNA sponges led to different physiological and pathological states in the two ceRNA networks. These lncRNAs could help provide biological insight in PC. The lncRNAs closely associated with PC may be potential therapeutic targets or molecular markers. Previous studies have shown that ceRNA expression level could be associated with patients’ survival. For example, lncRNA ROR, acted as a ceRNA of miR-145, could regulate NANOG expression level and was associated with poor survival of pancreatic cancer [44]. Moreover, the lncRNA HOTAIR, acted as a ceRNA of miR-331-3p, could regulate HER2 expression level and was associated with poor prognosis of gastric cancer [45]. We found that lncRNA MALAT1 in the gain ceRNA network was a hub node, and this lncRNA was a known PC-related lncRNA that could up-regulate EGR2 expression in PC patients, a key step in the pathogenesis of PC. We also identified an lncRNA MEG3 in the loss ceRNA network interacting with AQP3 and SYT1 to maintain homeostasis. MEG3 is highly expressed in healthy individuals, but its expression is lower in gastric cancer and NSCLC patients [46]. The expression of ceRNA pairs including these lncRNAs was significantly correlated with the overall survival of patients. More interestingly, we found that these patients showed a significant survival difference after 5 years, which might be due to the metastasis of PC. At last, we identified some drugs for treating PC by perturbing ceRNA pairs in gain and loss ceRNA networks. We further performed integrated analysis on CCEL, GDSC and TANRIC data for validating our predictions. Based on these data, we explored the relationship between the expression level of lncRNA/mRNA in ceRNA pair and chemoresponse of drug. Finally, we identified three drugs whose chemoresponse associated with the expression level of ceRNA pair, including Cisplatin, Cyclopamine and Paclitaxel. The ceRNA targeted by miRNA and the miRNA expression could be altered by small molecules, thus these small molecules might be used for cancer treatments. Small molecule drugs had many advantages, such as increased likelihood to be absorbed, and oral administration. Small molecule targeted therapy was one of the new approaches for cancer treatment. In this study, we investigated the potential of small molecule drugs to regulate two ceRNA networks.

We are only beginning to understand the mechanism by which lncRNAs perform their regulatory functions in ceRNA networks. Although our study has provided biological insights into the gain and loss ceRNA networks in PC, additional experiments will be required to further validate our findings. In addition, this computational framework can be easily extended to other cancer types or diseases, if the samples are simultaneously measured the mRNA, lncRNA and miRNA expression levels. We believed that with the increasing volume of multi-omics data, the accuracy and stability of our approach would be improved.

## MATERIALS AND METHODS

### Data collection and processing

We downloaded the prostate cancer gene and miRNA expression profiles (GSE21032) [47] from the Gene Expression Omnibus (GEO). The samples in which the expression level of both genes and miRNAs were measured were retained for the following analysis; these included 111 disease samples and 28 normal samples. All of the expression profiles were log2 transformed.

We used Du *et al*. [48] to re-annotate the probes from exon microarray and obtained the lncRNAs and mRNAs with at least four probes uniquely mapped to them. As a result, 4077 lncRNA genes had at least four probes covering their annotated exons, and the probe sets were mapped to Ensembl GeneIDs based on the Ensembl database (Homo sapiens GRCh38, release 81). We obtained the potential targeted relationships among lncRNAs, genes and miRNAs from the miRcode database (miRcode 11) [49] which provided ‘whole transcriptome’ human microRNA target predictions based on the comprehensive GENCODE gene annotation. As a result, we obtained 17,170 mRNAs, 374 miRNAs and 4,077 lncRNAs for this study. Additionally, we obtained 564 PC-specific tumor suppressor genes and lncRNAs from TSGene 2.0 database [50].

### Identification of highly expressed lncRNAs in PC

The high expression of oncogenes and low expression of tumor suppressor genes are important factors in cancer development. Recently, some studies have identified that lncRNAs can crosstalk with coding RNAs by competing for binding to shared microRNAs [51]. The high expression level of lncRNAs acting as miRNA sponges can weaken the inhibitory efficacy of miRNAs on gene expression [52]. To identify the lncRNAs with high level of expression, we computed the mean expression value of each lncRNA in cancer and normal samples and sorted the lncRNAs in descending order based on the mean expression value. Finally, we defined the top five percent lncRNAs as the highly expressed lncRNAs in cancer and normal samples.

### Identification of significant lncRNA-miRNA-mRNA triples

For a given pair of lncRNA-mRNA, *A* and *B*, there were *N_A_* miRNAs targeting *A* and *N_B_* miRNAs targeting *B*. Moreover, *A* and *B* were co-regulated by *n* miRNAs. If $\frac{n}{N_{A}+N_{B}-n}\geq 0.7$, this lncRNA-mRNA pair may be a potential ceRNA. There were 15,795,960 triads of lncRNAs, mRNAs and miRNAs satisfying this criterion. We further filtered these triples based upon the high level of expression of lncRNAs in cancer and normal samples. If the lncRNA of a given triple was a highly expressed lncRNA, the triple was included in the study. At last, we used the mutual information and CMI to calculate the statistical significance of triples according to

DI=I[miR;mRNA|lncRNA]−I[miR;mRNA]

where *I[miR;mRNA]* was the mutual information between miRNA and mRNA, *I[miR;mRNA|lncRNA]* was the mutual information between miRNA and mRNA under lncRNA condition. The higher Δ*I* value indicated that the lncRNA acted as a miRNA sponge with a higher efficacy on miRNA and gene interactions [18].

When the calculated three tags were consistent with the data of the sample, we obtained the real lncRNA mediating the Δ*I* values, permutated the samples of lncRNA tags 1000 times, and compared the real Δ*I* value with 1000 times random Δ*I* values to test the stability of the algorithm. We selected the mRNA, miRNA and lncRNA triples with *P* value <0.01 in the normal and cancer datasets by the Δ*I* method, and eventually found triples that complied with all of the above conditions in cancer and normal samples.

### Determination of the cancer and normal ceRNA network

Finally, the lncRNA-mRNA interaction network was determined for cancer and normal samples by assembling all significant triples identified above. A node represented an lncRNA or mRNA, and two nodes were interconnected if they significantly competed for miRNAs. Each ceRNA represents an lncRNA-mRNA pair, and each ceRNA pair may compete for several different miRNAs.

### Extraction of gain and loss ceRNA network

To determine the specific gain and loss relationship in cancer and normal networks, the combined interaction datasets were loaded into and visualized with the Cytoscape v2.8.3 [53]. Cytoscape was used to analyze the networks, and the ExprEssence plugin [54] was used to filter the interactions of the network. We condensed the network, highlighting those links across which the largest changes could be observed. By interactive use of ExprEssence, we only retained the 5% interactions that showed the largest differences in cancer and normal samples. Specifically, in the cancer network, we kept the edges in which the lncRNA and gene expression was higher in cancer than in normal samples; for the normal network, we just chose the opposite. We determined specific normal and cancer networks in which the lncRNA and mRNA interaction was specific in the cancer samples or in the normal samples. Nodes in the networks represented mRNAs and lncRNA, and the edges represented miRNAs mediating their interactions. lncRNAs acting as potential miRNA sponge were required to meet three conditions: i) lncRNA high expression, ii) sharing binding sites for miRNAs and iii) a statistically significant association.

### Survival analysis

In this study, we constructed two ceRNA networks for PC samples and normal prostate samples, and identified many loss and gain relationships in these ceRNA networks. We next investigated whether the loss and gain could distinguish PC patients with a good or poor outcome. Thus, we first obtained one PC dataset (GSE21032) from GEO database: this dataset had 111 patients with their mRNA and lncRNA expression and clinical information. Second, we used the *K*-means method (*K*=2) to cluster the 111 patients into two groups based on mRNA and lncRNA expression. Finally, the Kaplan–Meier curve and the log-rank test were used to evaluate the difference of overall survival time between the two patient groups.

## SUPPLEMENTARY MATERIALS FIGURES AND TABLES










