# Supplementary material for: The gain and loss of long noncoding RNA associated-competing endogenous RNAs in prostate cancer
Source: Oncotarget. 2016 Aug 9;7(35):57228–38. doi: 10.18632/oncotarget.11128 (PMC5302985; doi:10.18632/oncotarget.11128)
Supplement: Supplementary file 1 [file oncotarget-07-57228-s001.pdf]

The gain and loss of long noncoding RNA associated-competing endogenous RNAs in prostate cancer

SUPPLEMENTARY FIGURES AND TABLES

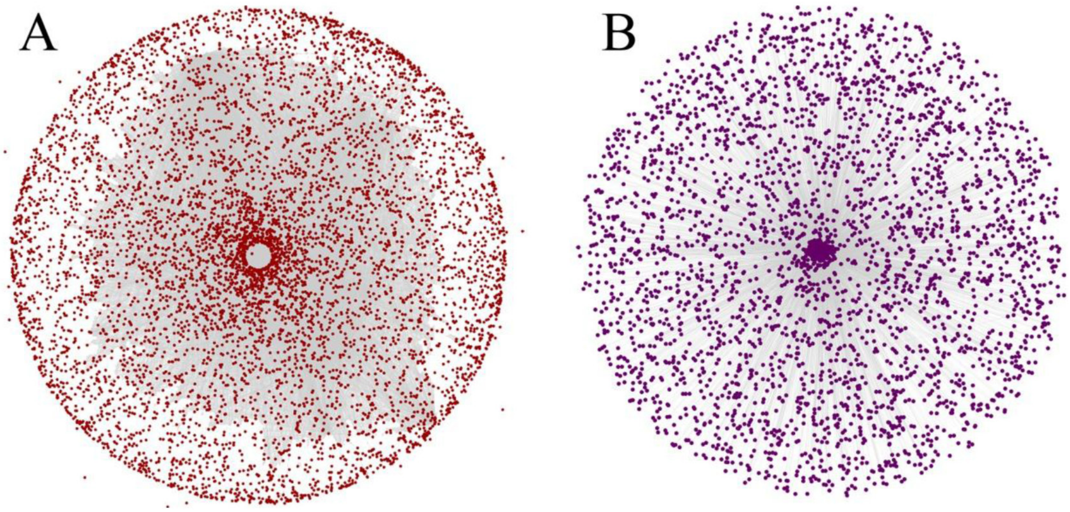

Supplementary Figure S1: Cancer and normal ceRNA networks. A. cancer ceRNAs network; **B.** normal ceRNAs network.

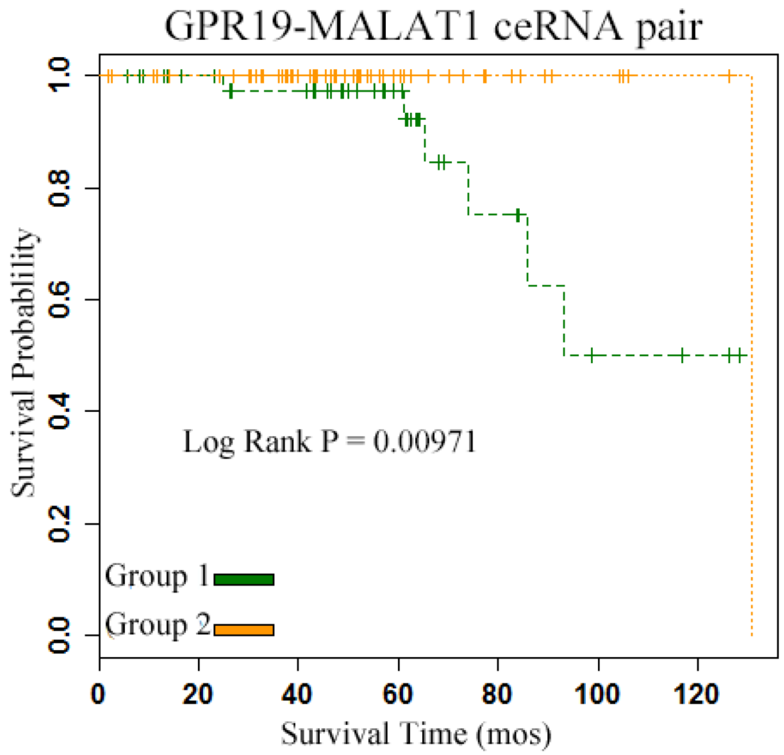

Supplementary Figure S2: ceRNA pairs were significantly correlated with the overall survival of PC patients in the gain ceRNA networks.

Supplementary Table S1: highly expressed lncRNAs in cancer and normal samples.

See Supplementary File 1

Supplementary Table S2: Gain ceRNA network.

See Supplementary File 2

Supplementary Table S3: Loss ceRNA network.

See Supplementary File 3

Supplementary Table S4: Tumor suppressor mRNAs and lncRNAs in loss ceRNA network

Gene name	Gene type
CAV1	protein_coding
IER3	protein_coding
VWA5A	protein_coding
ZNF185	protein_coding
VSNL1	protein_coding
ZBTB4	protein_coding
TNFRSF12A	protein_coding
IGFBP4	protein_coding
LIMA1	protein_coding
EFNA5	protein_coding
GJA1	protein_coding
MEG3	lncRNA

Supplementary Table S5: ceRNA pairs significantly correlated with overall survival of PC patients in gain network.

See Supplementary File 4

Supplementary Table S6: ceRNA pairs significantly correlatied with overall survival of PC patients in loss network

Log Rank P value	mRNA	miRNA	lncRNA
0.00297	MBTPS1	hsa-miR-22	MEG3 [13]
0.00364	AQP3 [14]	hsa-miR-204	MEG3
0.00585	STOX2	hsa-miR-590-5p	MEG3
0.0137	PPARGC1A	hsa-miR-206	MEG3
0.0137	PPARGC1A	hsa-miR-222	MEG3
0.0149	GLTP	hsa-miR-7	MEG3
0.0255	DUOX1	hsa-miR-140-5p	MEG3
0.0294	CYBRD1	hsa-miR-143	MEG3
0.0331	PPFIBP1	hsa-miR-23a	MEG3
0.0331	PPFIBP1	hsa-miR-34a	MEG3
0.0331	PPFIBP1	hsa-miR-132	MEG3
0.0331	PPFIBP1	hsa-miR-10a	MEG3
0.0331	PPFIBP1	hsa-miR-19b	MEG3
0.0333	CNN1	hsa-miR-15a	RP11-475O6.1
0.0405	UST	hsa-miR-7	MEG3
0.0477	SYT1	hsa-miR-206	MEG3

Supplementary Table S7: Small molecules drug regulate miRNA expression

miRNA	Small Melocule	Expression pattern of miRNA
has-miR-200c [15-17]	Paclitaxel+Cyclopamine	up-regulated [18]
has-miR-200c	17beta-estradiol (E2)	up-regulated [19]
has-miR-205 [16, 20-22]	1,2,6-Tri-O-galloyl-beta-D-glucopyranose (TGGP)	up-regulated [23]
has-miR-200a [24]	17beta-estradiol (E2)	up-regulated [25]
has-miR-106a [26]	17beta-estradiol (E2)	up-regulated [27]
has-miR-200c	Reversine	up-regulated [28]
has-miR-106a	Cisplatin	down-regulated [29]
has-miR-16	Estrogen	down-regulated [30]
has-miR-27a [31, 32]	Budesonide	down-regulated [33]
has-miR-27a	17beta-estradiol (E2)	down-regulated [25]
has-miR-27a	1,2,6-Tri-O-galloyl-beta-D-glucopyranose (TGGP)	down-regulated [23]
has-miR-27a	Genistein	down-regulated [34]
has-miR-150	1,2,6-Tri-O-galloyl-beta-D-glucopyranose (TGGP)	down-regulated [23]
has-miR-150	Cisplatin	down-regulated [35]

REFERENCES

1. Ren S, Liu Y, Xu W, Sun Y, Lu J, Wang F, Wei M, Shen J, Hou J, Gao X, Xu C, Huang J, Zhao Y and Sun Y. Long noncoding RNA MALAT-1 is a new potential therapeutic target for castration resistant prostate cancer. The Journal of urology. 2013; 190:2278-2287.
2. Scaggiante B, Dapas B, Bonin S, Grassi M, Zennaro C, Farra R, Cristiano L, Siracusano S, Zanconati F, Giansante C and Grassi G. Dissecting the expression of EEF1A1/2 genes in human prostate cancer cells: the potential of EEF1A2 as a hallmark for prostate transformation and progression. British journal of cancer. 2012; 106:166-173.
3. LaTulippe E, Satagopan J, Smith A, Scher H, Scardino P, Reuter V and Gerald WL. Comprehensive gene expression analysis of prostate cancer reveals distinct transcriptional programs associated with metastatic disease. Cancer research. 2002; 62:4499-4506.
4. Montpetit A and Sinnett D. Physical mapping of the G-protein coupled receptor 19 (GPR19) in the chromosome 12p12.3 region frequently rearranged in cancer cells. Human genetics. 1999; 105:162-164.
5. Lin JF, Xu J, Tian HY, Gao X, Chen QX, Gu Q, Xu GJ, Song JD and Zhao FK. Identification of candidate prostate cancer biomarkers in prostate needle biopsy specimens using proteomic analysis. International journal of cancer Journal international du cancer. 2007; 121:2596-2605.
6. Pflueger D, Terry S, Sboner A, Habegger L, Esgueva R, Lin PC, Svensson MA, Kitabayashi N, Moss BJ, MacDonald TY, Cao X, Barrette T, Tewari AK, Chee MS, Chinnaiyan AM, Rickman DS, et al. Discovery of non-ETS gene fusions in human prostate cancer using next-generation RNA sequencing. Genome research. 2011; 21:56-67.
7. Barbieri CE, Baca SC, Lawrence MS, Demichelis F, Blattner M, Theurillat JP, White TA, Stojanov P, Van Allen E, Stransky N, Nickerson E, Chae SS, Boysen G, Auclair D, Onofrio RC, Park K, et al. Exome sequencing identifies recurrent SPOP, FOXA1 and MED12 mutations in prostate cancer. Nature genetics. 2012; 44:685-689.
8. Chung TD, Yu JJ, Spiotto MT, Bartkowski M and Simons JW. Characterization of the role of IL-6 in the progression of prostate cancer. Prostate. 1999; 38:199-207.
9. Yeh HY, Cheng SW, Lin YC, Yeh CY, Lin SF and Soo VW. Identifying significant genetic regulatory networks in the prostate cancer from microarray data based on transcription factor analysis and conditional independency. BMC medical genomics. 2009; 2:70.
10. Gu X, Zerbini LF, Otu HH, Bhasin M, Yang Q, Joseph MG, Grall F, Onatunde T, Correa RG and Libermann TA. Reduced PDEF expression increases invasion and expression of mesenchymal genes in prostate cancer cells. Cancer research. 2007; 67:4219-4226.
11. de Boussac H, Pommier AJ, Dufour J, Trousson A, Caira F, Volle DH, Baron S and Lobaccaro JM. LXR, prostate cancer and cholesterol: the Good, the Bad and the Ugly. American journal of cancer research. 2013; 3:58-69.
12. Zeng Y, He Y, Yang F, Mooney SM, Getzenberg RH, Orban J and Kulkarni P. The cancer/testis antigen prostate-associated gene 4 (PAGE4) is a highly intrinsically disordered protein. The Journal of biological chemistry. 2011; 286:13985-13994.
13. Luo G, Wang M, Wu X, Tao D, Xiao X, Wang L, Min F, Zeng F and Jiang G. Long Non-Coding RNA MEG3 Inhibits Cell Proliferation and Induces Apoptosis in Prostate Cancer. Cellular physiology and biochemistry : international journal of experimental cellular physiology, biochemistry, and pharmacology. 2015; 37:2209-2220.
14. Pei L, Yang G, Jiang J, Jiang R, Deng Q, Chen B and Gan X. Expression of aquaporins in prostate and seminal vesicles of diabetic rats. The journal of sexual medicine. 2013; 10:2975-2985.
15. Vallejo DM, Caparros E and Dominguez M. Targeting Notch signalling by the conserved miR-8/200 microRNA family in development and cancer cells. The EMBO journal. 2011; 30:756-769.
16. Tang X, Tang X, Gal J, Kyprianou N, Zhu H and Tang G. Detection of microRNAs in prostate cancer cells by microRNA array. Methods in molecular biology. 2011; 732:69-88.
17. Liu YN, Yin JJ, Abou-Kheir W, Hynes PG, Casey OM, Fang L, Yi M, Stephens RM, Seng V, Sheppard-Tillman H, Martin P and Kelly K. MiR-1 and miR-200 inhibit EMT via Slug-dependent and tumorigenesis via Slug-independent mechanisms. Oncogene. 2013; 32:296-306.
18. Singh S, Chitkara D, Mehrazin R, Behrman SW, Wake RW and Mahato RI. Chemoresistance in prostate cancer cells is regulated by miRNAs and Hedgehog pathway. PloS one. 2012; 7:e40021.
19. Shah MY, Pan X, Fix LN, Farwell MA and Zhang B. 5-Fluorouracil drug alters the microRNA expression profiles in MCF-7 breast cancer cells. Journal of cellular physiology. 2011; 226:1868-1878.
20. Gandellini P, Folini M, Longoni N, Pennati M, Binda M, Colecchia M, Salvioni R, Supino R, Moretti R, Limonta P, Valdagni R, Daidone MG and Zaffaroni N. miR-205 Exerts tumor-suppressive functions in human prostate through down-regulation of protein kinase Cepsilon. Cancer research. 2009; 69:2287-2295.
21. Hulf T, Sibbritt T, Wiklund ED, Bert S, Strbenac D, Statham AL, Robinson MD and Clark SJ. Discovery pipeline for epigenetically deregulated miRNAs in cancer: integration of primary miRNA transcription. BMC genomics. 2011; 12:54.

22. Watahiki A, Wang Y, Morris J, Dennis K, O'Dwyer HM, Gleave M, Gout PW and Wang Y. MicroRNAs associated with metastatic prostate cancer. PloS one. 2011; 6:e24950.
23. Ai RT, Wu SY, Wen XY, Xu W, Lv L and Wu SG. [A new natural polyphenol BJA32531 inhibited the proliferation and regulated miRNA expression in human HepG2 hepatocarcinoma cells]. Zhong yao cai = Zhongyaocai = Journal of Chinese medicinal materials. 2011; 34:1734-1740.
24. Barron N, Keenan J, Gammell P, Martinez VG, Freeman A, Masters JR and Clynes M. Biochemical relapse following radical prostatectomy and miR-200a levels in prostate cancer. Prostate. 2012; 72:1193-1199.
25. Bhat-Nakshatri P, Wang G, Collins NR, Thomson MJ, Geistlinger TR, Carroll JS, Brown M, Hammond S, Srour EF, Liu Y and Nakshatri H. Estradiol-regulated microRNAs control estradiol response in breast cancer cells. Nucleic acids research. 2009; 37:4850-4861.
26. Lum AM, Wang BB, Li L, Channa N, Bartha G and Wabl M. Retroviral activation of the mir-106a microRNA cistron in T lymphoma. Retrovirology. 2007; 4:5.
27. Kovalchuk O, Tryndyak VP, Montgomery B, Boyko A, Kutanzi K, Zemp F, Warbritton AR, Latendresse JR, Kovalchuk I, Beland FA and Pogribny IP. Estrogen-induced rat breast carcinogenesis is characterized by alterations in DNA methylation, histone modifications and aberrant microRNA expression. Cell Cycle. 2007; 6:2010-2018.
28. Kim M, Yi SA, Lee H, Bang SY, Park EK, Lee MG, Nam KH, Yoo JH, Lee DH, Ryu HW, Kwon SH and Han JW. Reversine induces multipotency of lineage-committed cells through epigenetic silencing of miR-133a. Biochemical and biophysical research communications. 2014; 445:255-262.
29. Boren T, Xiong Y, Hakam A, Wenham R, Apte S, Chan G, Kamath SG, Chen DT, Dressman H and Lancaster JM. MicroRNAs and their target messenger RNAs associated with ovarian cancer response to chemotherapy. Gynecologic oncology. 2009; 113:249-255.
30. Yu X, Zhang X, Dhakal IB, Beggs M, Kadlubar S and Luo D. Induction of cell proliferation and survival genes by estradiol-repressed microRNAs in breast cancer cells. BMC cancer. 2012; 12:29.
31. Epis MR, Giles KM, Barker A, Kendrick TS and Leedman PJ. miR-331-3p regulates ERBB-2 expression and androgen receptor signaling in prostate cancer. The Journal of biological chemistry. 2009; 284:24696-24704.
32. Fletcher CE, Dart DA, Sita-Lumsden A, Cheng H, Rennie PS and Bevan CL. Androgen-regulated processing of the oncomir miR-27a, which targets Prohibitin in prostate cancer. Human molecular genetics. 2012; 21:3112-3127.
33. Izzotti A, Larghero P, Cartiglia C, Longobardi M, Pfeffer U, Steele VE and De Flora S. Modulation of microRNA expression by budesonide, phenethyl isothiocyanate and cigarette smoke in mouse liver and lung. Carcinogenesis. 2010; 31:894-901.
34. Sun Q, Cong R, Yan H, Gu H, Zeng Y, Liu N, Chen J and Wang B. Genistein inhibits growth of human uveal melanoma cells and affects microRNA-27a and target gene expression. Oncology reports. 2009; 22:563-567.
35. Xie SY, Li YJ, Wang PY, Jiao F, Zhang S and Zhang WJ. miRNA-regulated expression of oncogenes and tumor suppressor genes in the cisplatin-inhibited growth of K562 cells. Oncology reports. 2010; 23:1693-1700.
