# Supplementary material for: The gain and loss of long noncoding RNA associated-competing endogenous RNAs in prostate cancer
Source: Oncotarget. 2016 Aug 9;7(35):57228–38. doi: 10.18632/oncotarget.11128 (PMC5302985; doi:10.18632/oncotarget.11128)
Supplement: Supplementary file 2 [file oncotarget-07-57228-s002.docx]

**Table S1 highly expressed lncRNAs in cancer and normal samples**

| Cancer highly expressed lncRNAs | Normal highly expressed lncRNAs |
| --- | --- |
| ENSG00000251562 | ENSG00000251562 |
| ENSG00000260682 | ENSG00000260682 |
| ENSG00000245532 | ENSG00000229370 |
| ENSG00000231485 | ENSG00000231485 |
| ENSG00000224532 | ENSG00000245532 |
| ENSG00000229370 | ENSG00000224532 |
| ENSG00000229066 | ENSG00000224460 |
| ENSG00000228826 | ENSG00000258909 |
| ENSG00000224460 | ENSG00000232388 |
| ENSG00000258909 | ENSG00000229066 |
| ENSG00000212694 | ENSG00000228826 |
| ENSG00000231689 | ENSG00000212694 |
| ENSG00000250427 | ENSG00000227082 |
| ENSG00000227082 | ENSG00000233008 |
| ENSG00000250312 | ENSG00000250427 |
| ENSG00000232388 | ENSG00000253864 |
| ENSG00000256955 | ENSG00000228105 |
| ENSG00000228105 | ENSG00000256955 |
| ENSG00000233008 | ENSG00000231689 |
| ENSG00000259681 | ENSG00000250312 |
| ENSG00000223573 | ENSG00000249669 |
| ENSG00000228527 | ENSG00000223573 |
| ENSG00000258654 | ENSG00000248125 |
| ENSG00000261423 | ENSG00000258654 |
| ENSG00000248125 | ENSG00000261423 |
| ENSG00000203279 | ENSG00000259681 |
| ENSG00000185044 | ENSG00000203279 |
| ENSG00000227150 | ENSG00000227150 |
| ENSG00000249621 | ENSG00000185044 |
| ENSG00000179523 | ENSG00000249621 |
| ENSG00000214837 | ENSG00000230937 |
| ENSG00000226328 | ENSG00000226328 |
| ENSG00000225860 | ENSG00000179523 |
| ENSG00000238035 | ENSG00000228527 |
| ENSG00000253678 | ENSG00000253678 |
| ENSG00000170846 | ENSG00000214837 |
| ENSG00000234035 | ENSG00000170846 |
| ENSG00000226208 | ENSG00000231566 |
| ENSG00000233064 | ENSG00000234035 |
| ENSG00000231566 | ENSG00000238035 |
| ENSG00000232100 | ENSG00000231437 |
| ENSG00000249736 | ENSG00000260517 |
| ENSG00000230945 | ENSG00000233064 |
| ENSG00000231437 | ENSG00000223749 |
| ENSG00000231560 | ENSG00000231560 |
| ENSG00000260517 | ENSG00000255021 |
| ENSG00000232019 | ENSG00000249395 |
| ENSG00000228952 | ENSG00000262223 |
| ENSG00000248551 | ENSG00000232019 |
| ENSG00000255021 | ENSG00000228952 |
| ENSG00000176320 | ENSG00000223704 |
| ENSG00000251015 | ENSG00000233516 |
| ENSG00000243629 | ENSG00000255328 |
| ENSG00000255328 | ENSG00000232224 |
| ENSG00000238142 | ENSG00000238142 |
| ENSG00000223704 | ENSG00000232855 |
| ENSG00000223749 | ENSG00000237807 |
| ENSG00000237940 | ENSG00000249736 |
| ENSG00000255094 | ENSG00000250149 |
| ENSG00000249790 | ENSG00000178947 |
| ENSG00000257595 | ENSG00000237940 |
| ENSG00000260404 | ENSG00000225860 |
| ENSG00000247095 | ENSG00000260854 |
| ENSG00000249395 | ENSG00000251015 |
| ENSG00000233261 | ENSG00000226208 |
| ENSG00000262223 | ENSG00000255094 |
| ENSG00000258676 | ENSG00000225857 |
| ENSG00000260854 | ENSG00000247095 |
| ENSG00000232224 | ENSG00000262165 |
| ENSG00000248740 | ENSG00000260404 |
| ENSG00000178947 | ENSG00000258676 |
| ENSG00000229774 | ENSG00000233261 |
| ENSG00000226115 | ENSG00000225285 |
| ENSG00000250149 | ENSG00000232100 |
| ENSG00000258551 | ENSG00000249790 |
| ENSG00000251331 | ENSG00000257595 |
| ENSG00000227066 | ENSG00000176320 |
| ENSG00000259108 | ENSG00000259672 |
| ENSG00000225857 | ENSG00000259758 |
| ENSG00000248431 | ENSG00000226115 |
| ENSG00000225285 | ENSG00000248551 |
| ENSG00000262343 | ENSG00000229774 |
| ENSG00000228235 | ENSG00000230590 |
| ENSG00000231983 | ENSG00000255778 |
| ENSG00000255778 | ENSG00000251321 |
| ENSG00000262179 | ENSG00000215386 |
| ENSG00000262165 | ENSG00000259108 |
| ENSG00000231346 | ENSG00000230945 |
| ENSG00000251292 | ENSG00000231346 |
| ENSG00000254885 | ENSG00000228235 |
| ENSG00000249669 | ENSG00000248740 |
| ENSG00000249430 | ENSG00000214548 |
| ENSG00000254869 | ENSG00000262343 |
| ENSG00000224023 | ENSG00000254885 |
| ENSG00000251230 | ENSG00000232347 |
| ENSG00000233516 | ENSG00000254869 |
| ENSG00000232347 | ENSG00000262179 |
| ENSG00000259672 | ENSG00000227066 |
| ENSG00000205015 | ENSG00000231983 |
| ENSG00000215908 | ENSG00000243629 |
| ENSG00000236507 | ENSG00000258551 |
| ENSG00000229236 | ENSG00000224023 |
| ENSG00000253864 | ENSG00000255193 |
| ENSG00000233864 | ENSG00000251331 |
| ENSG00000188185 | ENSG00000229236 |
| ENSG00000232233 | ENSG00000249430 |
| ENSG00000260260 | ENSG00000188185 |
| ENSG00000254242 | ENSG00000215908 |
| ENSG00000263154 | ENSG00000205015 |
| ENSG00000240219 | ENSG00000249740 |
| ENSG00000226497 | ENSG00000236507 |
| ENSG00000230590 | ENSG00000233864 |
| ENSG00000245750 | ENSG00000251230 |
| ENSG00000225598 | ENSG00000234689 |
| ENSG00000255193 | ENSG00000236537 |
| ENSG00000225872 | ENSG00000240219 |
| ENSG00000231476 | ENSG00000263154 |
| ENSG00000231507 | ENSG00000232233 |
| ENSG00000249740 | ENSG00000251292 |
| ENSG00000196421 | ENSG00000239407 |
| ENSG00000185904 | ENSG00000231507 |
| ENSG00000185168 | ENSG00000225872 |
| ENSG00000261095 | ENSG00000196421 |
| ENSG00000234689 | ENSG00000260785 |
| ENSG00000254002 | ENSG00000100181 |
| ENSG00000260785 | ENSG00000185904 |
| ENSG00000235152 | ENSG00000185168 |
| ENSG00000259758 | ENSG00000261244 |
| ENSG00000204583 | ENSG00000259275 |
| ENSG00000236537 | ENSG00000253364 |
| ENSG00000240567 | ENSG00000233137 |
| ENSG00000259275 | ENSG00000248431 |
| ENSG00000233214 | ENSG00000228709 |
| ENSG00000227373 | ENSG00000229719 |
| ENSG00000261020 | ENSG00000260802 |
| ENSG00000259820 | ENSG00000232655 |
| ENSG00000253364 | ENSG00000231476 |
| ENSG00000261804 | ENSG00000186594 |
| ENSG00000250198 | ENSG00000225598 |
| ENSG00000205293 | ENSG00000260396 |
| ENSG00000233137 | ENSG00000235152 |
| ENSG00000223823 | ENSG00000259820 |
| ENSG00000261244 | ENSG00000230563 |
| ENSG00000250971 | ENSG00000226380 |
| ENSG00000215866 | ENSG00000226497 |
| ENSG00000228709 | ENSG00000255983 |
| ENSG00000262089 | ENSG00000223823 |
| ENSG00000232655 | ENSG00000261095 |
| ENSG00000262454 | ENSG00000224032 |
| ENSG00000259124 | ENSG00000254002 |
| ENSG00000251471 | ENSG00000227718 |
| ENSG00000229719 | ENSG00000215560 |
| ENSG00000255983 | ENSG00000215866 |
| ENSG00000239407 | ENSG00000226137 |
| ENSG00000100181 | ENSG00000240567 |
| ENSG00000237359 | ENSG00000272168 |
| ENSG00000226137 | ENSG00000262089 |
| ENSG00000215560 | ENSG00000253821 |
| ENSG00000242407 | ENSG00000262454 |
| ENSG00000260492 | ENSG00000251377 |
| ENSG00000203635 | ENSG00000237359 |
| ENSG00000260896 | ENSG00000261804 |
| ENSG00000235215 | ENSG00000260260 |
| ENSG00000236983 | ENSG00000259124 |
| ENSG00000253821 | ENSG00000259721 |
| ENSG00000259721 | ENSG00000233694 |
| ENSG00000186594 | ENSG00000261020 |
| ENSG00000228437 | ENSG00000250198 |
| ENSG00000231428 | ENSG00000250971 |
| ENSG00000251377 | ENSG00000205293 |
| ENSG00000233694 | ENSG00000262370 |
| ENSG00000234977 | ENSG00000234977 |
| ENSG00000258969 | ENSG00000236983 |
| ENSG00000237807 | ENSG00000227373 |
| ENSG00000224592 | ENSG00000224592 |
| ENSG00000250934 | ENSG00000228437 |
| ENSG00000237590 | ENSG00000230699 |
| ENSG00000262370 | ENSG00000231428 |
| ENSG00000230563 | ENSG00000260492 |
| ENSG00000226041 | ENSG00000224481 |
| ENSG00000230699 | ENSG00000254242 |
| ENSG00000234546 | ENSG00000250934 |
| ENSG00000226380 | ENSG00000234546 |
| ENSG00000234265 | ENSG00000228222 |
| ENSG00000214548 | ENSG00000242407 |
| ENSG00000227718 | ENSG00000204754 |
| ENSG00000230937 | ENSG00000180712 |
| ENSG00000258048 | ENSG00000223546 |
| ENSG00000204754 | ENSG00000230844 |
| ENSG00000224790 | ENSG00000258969 |
| ENSG00000260792 | ENSG00000176840 |
| ENSG00000225554 | ENSG00000237590 |
| ENSG00000236081 | ENSG00000255455 |
| ENSG00000230844 | ENSG00000258048 |
| ENSG00000226091 | ENSG00000242474 |
| ENSG00000227418 | ENSG00000234265 |
| ENSG00000251321 | ENSG00000235215 |
| ENSG00000227363 | ENSG00000204583 |
| ENSG00000235304 | ENSG00000226041 |
| ENSG00000237461 | ENSG00000233901 |
