# Supplementary material for: The gain and loss of long noncoding RNA associated-competing endogenous RNAs in prostate cancer
Source: Oncotarget. 2016 Aug 9;7(35):57228–38. doi: 10.18632/oncotarget.11128 (PMC5302985; doi:10.18632/oncotarget.11128)
Supplement: Supplementary file 3 [file oncotarget-07-57228-s003.docx]

**Table S2. Gain ceRNA network**

| **mRNA** | **miRNA** | **lncRNA** |
| --- | --- | --- |
| AADAT | hsa-miR-16 | LINC00937 |
| AAMP | hsa-miR-24 | RP11-1080G15.2 |
| ABCC4 | hsa-miR-132 | AC083843.1 |
| ABCC4 | hsa-miR-23a | AC083843.1 |
| ABCC4 | hsa-miR-141 | MEG3 |
| ACP5 | hsa-miR-92a | RP11-1018N14.2 |
| ACRV1 | hsa-miR-383 | NEAT1 |
| ACSM1 | hsa-miR-96 | NEAT1 |
| ACSM2B | hsa-miR-30a | MALAT1 |
| ACSM2B | hsa-miR-181b | MALAT1 |
| ADAMTS3 | hsa-miR-101 | MALAT1 |
| ADAMTS3 | hsa-miR-204 | MALAT1 |
| ADAMTS3 | hsa-miR-144 | MALAT1 |
| ADRB2 | hsa-miR-7 | NEAT1 |
| ADRB2 | hsa-miR-497 | RP11-1007O24.3 |
| AICDA | hsa-miR-181b | MALAT1 |
| AICDA | hsa-miR-215 | MALAT1 |
| ALCAM | hsa-miR-152 | MALAT1 |
| ALDH1A3 | hsa-miR-205 | AC083843.1 |
| ALDH1A3 | hsa-miR-30a | NEAT1 |
| ALDH1A3 | hsa-miR-200c | NEAT1 |
| ALOX12 | hsa-miR-424 | RP11-982M15.8 |
| ALS2CR11 | hsa-miR-181d | RP11-1018N14.2 |
| ALS2CR11 | hsa-miR-30e | RP11-1018N14.2 |
| ANAPC11 | hsa-miR-31 | RP11-384K6.6 |
| ANKRD24 | hsa-miR-199b-5p | RP11-1018N14.2 |
| ANKRD24 | hsa-miR-27b | RP11-1018N14.2 |
| ANKRD55 | hsa-miR-93 | AC144450.2 |
| AP1M2 | hsa-miR-150 | PRCAT47 |
| APOF | hsa-miR-128 | LINC00202-1 |
| APOF | hsa-miR-27b | LINC01089 |
| ARFIP2 | hsa-miR-128 | TINCR |
| ARRDC1 | hsa-miR-15b | AC144450.2 |
| ARRDC1-AS1 | hsa-miR-10a | RP3-473L9.4 |
| ATF3 | hsa-miR-27b | LINC01281 |
| ATF3 | hsa-miR-222 | LINC01281 |
| ATN1 | hsa-miR-15b | AC144450.2 |
| ATP8A1 | hsa-miR-30a | NEAT1 |
| ATP8A1 | hsa-miR-15a | NEAT1 |
| AVPR2 | hsa-miR-27b | RP11-744K17.1 |
| B9D1 | hsa-miR-10b | RP11-982M15.8 |
| BAMBI | hsa-miR-17 | TTTY15 |
| BICD1 | hsa-miR-181b | MALAT1 |
| BICD1 | hsa-miR-497 | TPTEP1 |
| BICD1 | hsa-miR-499-5p | TPTEP1 |
| BICD1 | hsa-miR-200c | MALAT1 |
| BICD1 | hsa-miR-194 | MALAT1 |
| BMPR1B | hsa-miR-200c | NEAT1 |
| BMPR1B | hsa-miR-150 | NEAT1 |
| BUB1 | hsa-miR-20a | RP11-287F9.2 |
| C12orf56 | hsa-miR-181d | RP11-982M15.8 |
| C19orf38 | hsa-miR-125a-5p | LINC01602 |
| C19orf52 | hsa-miR-129-5p | DRAIC |
| C1orf61 | hsa-miR-143 | RP11-152L7.2 |
| C1QL4 | hsa-miR-10a | RP11-982M15.8 |
| C2orf72 | hsa-miR-215 | MALAT1 |
| C2orf72 | hsa-miR-205 | MALAT1 |
| C2orf72 | hsa-miR-200b | MALAT1 |
| C4BPB | hsa-miR-146a | RP11-982M15.8 |
| C4orf46 | hsa-miR-218 | MALAT1 |
| C8orf4 | hsa-miR-203 | LINC01422 |
| C9orf152 | hsa-miR-150 | NEAT1 |
| C9orf78 | hsa-miR-129-5p | AC144450.2 |
| CA1 | hsa-miR-18b | AC007365.1 |
| CA2 | hsa-miR-106a | LINC00937 |
| CA5B | hsa-miR-152 | MALAT1 |
| CACNA1D | hsa-miR-203 | NEAT1 |
| CADM1 | hsa-miR-106a | NEAT1 |
| CAMKK2 | hsa-miR-30a | NEAT1 |
| CAMKK2 | hsa-miR-182 | NEAT1 |
| CAMKK2 | hsa-miR-30b | NEAT1 |
| CAMKK2 | hsa-miR-18b | NEAT1 |
| CCDC102B | hsa-miR-129-5p | RP11-152L7.2 |
| CCDC58 | hsa-miR-24 | RP11-152L7.2 |
| CCL11 | hsa-miR-33b | AC096649.3 |
| CD163 | hsa-miR-181d | RP11-744K17.1 |
| CD180 | hsa-miR-195 | RP11-982M15.8 |
| CD180 | hsa-miR-15a | RP11-982M15.8 |
| CD63 | hsa-miR-15b | AC144450.2 |
| CD80 | hsa-miR-21 | MALAT1 |
| CDC25C | hsa-miR-15b | RP11-982M15.8 |
| CDC37 | hsa-miR-34a | DRAIC |
| CDC6 | hsa-miR-363 | AC093642.3 |
| CDCA3 | hsa-miR-93 | RP11-99H8.1 |
| CDKN3 | hsa-miR-32 | MALAT1 |
| CECR2 | hsa-miR-93 | NEAT1 |
| CENPL | hsa-miR-129-5p | EIF3J-AS1 |
| CENPV | hsa-miR-181a | LINC00880 |
| CEP250 | hsa-miR-125a-5p | AC144450.2 |
| CER1 | hsa-miR-125a-5p | AC018866.1 |
| CER1 | hsa-miR-26a | AC018866.1 |
| CIDEC | hsa-miR-29b | RP11-982M15.8 |
| CLDN3 | hsa-miR-128 | LINC01281 |
| CLDN8 | hsa-miR-129-5p | NEAT1 |
| CLSPN | hsa-miR-150 | TINCR |
| CNTNAP2 | hsa-miR-200b | MALAT1 |
| CNTNAP2 | hsa-miR-150 | MALAT1 |
| CNTNAP2 | hsa-miR-30b | MALAT1 |
| CNTNAP2 | hsa-miR-152 | MALAT1 |
| CNTNAP2 | hsa-miR-200c | MALAT1 |
| COG7 | hsa-miR-93 | AC144450.2 |
| COL28A1 | hsa-miR-140-5p | LINC01422 |
| COX16 | hsa-miR-363 | RP11-982M15.8 |
| CPNE4 | hsa-miR-144 | NEAT1 |
| CPNE4 | hsa-miR-218 | FTX |
| CRYGC | hsa-miR-142-3p | RP11-1018N14.2 |
| CSDE1 | hsa-miR-106b | PRCAT47 |
| CSNK1G2-AS1 | hsa-miR-150 | RP11-83M16.5 |
| CSTL1 | hsa-miR-152 | RP11-143A12.3 |
| CTHRC1 | hsa-miR-30e | DRAIC |
| CTHRC1 | hsa-miR-30b | RP11-152L7.2 |
| CXCL10 | hsa-miR-135a | TPTEP1 |
| CXCL9 | hsa-miR-181b | MALAT1 |
| CXCL9 | hsa-miR-206 | MALAT1 |
| CYP2C9 | hsa-miR-15b | RP11-384K6.6 |
| CYP7A1 | hsa-miR-181d | RP11-982M15.8 |
| DAZ2 | hsa-miR-196a | NEAT1 |
| DBF4 | hsa-miR-140-5p | LINC01422 |
| DBF4 | hsa-miR-20b | MALAT1 |
| DDAH2 | hsa-miR-150 | PRCAT47 |
| DDIAS | hsa-miR-192 | MALAT1 |
| DEFB121 | hsa-miR-7 | CASC17 |
| DGCR6 | hsa-miR-195 | AC144450.2 |
| DIO3 | hsa-miR-199b-5p | RP11-1018N14.2 |
| DLX1 | hsa-miR-30a | DRAIC |
| DNAJC28 | hsa-miR-30a | RP11-160H22.5 |
| DOPEY2 | hsa-miR-129-5p | TINCR |
| DUSP21 | hsa-miR-26b | RP3-340N1.2 |
| EBF2 | hsa-miR-181d | NEAT1 |
| EBF2 | hsa-miR-33a | NEAT1 |
| ECT2 | hsa-miR-182 | AC083843.1 |
| ECT2 | hsa-miR-205 | NEAT1 |
| ECT2 | hsa-miR-205 | AC083843.1 |
| EDC4 | hsa-miR-15b | AC144450.2 |
| EDEM3 | hsa-miR-25 | AC083843.1 |
| EEF1A2 | hsa-miR-31 | RP11-384K6.6 |
| EGF | hsa-miR-145 | LINC00937 |
| EGR2 | hsa-miR-93 | MALAT1 |
| EGR2 | hsa-miR-129-5p | MALAT1 |
| ELAVL2 | hsa-miR-106b | AC083843.1 |
| EML2 | hsa-miR-338-3p | LINC01507 |
| ERGIC1 | hsa-miR-22 | NEAT1 |
| ESRRG | hsa-miR-200c | MALAT1 |
| EZH2 | hsa-miR-15a | NEAT1 |
| EZH2 | hsa-miR-92a | LINC01422 |
| F2RL1 | hsa-miR-26a | AC083843.1 |
| F7 | hsa-miR-10a | RP11-982M15.8 |
| FAM101A | hsa-miR-497 | AC144450.2 |
| FAM111A | hsa-miR-32 | RP11-982M15.8 |
| FAM111A | hsa-miR-15b | RP11-982M15.8 |
| FAM129B | hsa-miR-20b | AC144450.2 |
| FAM171A2 | hsa-miR-15a | LINC01507 |
| FAM171A2 | hsa-miR-16 | LINC01507 |
| FAM92B | hsa-miR-218 | LINC01602 |
| FAM96A | hsa-miR-212 | NEAT1 |
| FAT1 | hsa-miR-26a | PCGEM1 |
| FCRL2 | hsa-miR-363 | LINC00937 |
| FEV | hsa-miR-106a | RP11-99H8.1 |
| FLT3 | hsa-miR-22 | LINC00937 |
| FLT3 | hsa-miR-193b | LINC00937 |
| FLT3 | hsa-miR-96 | LINC00937 |
| FMN1 | hsa-miR-338-3p | MALAT1 |
| FMN1 | hsa-miR-205 | MALAT1 |
| FMN1 | hsa-miR-15b | MALAT1 |
| FMN1 | hsa-miR-181b | MALAT1 |
| FMOD | hsa-miR-23b | MALAT1 |
| FOLH1 | hsa-miR-200c | NEAT1 |
| FOXA1 | hsa-miR-93 | AC091814.3 |
| FOXA1 | hsa-miR-30d | AC091814.3 |
| FSHR | hsa-miR-33a | LINC00937 |
| FZD5 | hsa-miR-182 | MALAT1 |
| GBP5 | hsa-miR-338-3p | MALAT1 |
| GBP5 | hsa-miR-218 | MALAT1 |
| GCNT1 | hsa-miR-128 | AC083843.1 |
| GCNT1 | hsa-miR-363 | MALAT1 |
| GDF15 | hsa-miR-128 | LINC00614 |
| GDF15 | hsa-miR-128 | TTTY5 |
| GDPD1 | hsa-miR-200c | MALAT1 |
| GFPT1 | hsa-miR-214 | MALAT1 |
| GFPT1 | hsa-miR-218 | MALAT1 |
| GJB7 | hsa-miR-150 | TINCR |
| GLDC | hsa-miR-30d | DRAIC |
| GNE | hsa-miR-129-5p | MALAT1 |
| GNPNAT1 | hsa-miR-106a | TPTEP1 |
| GOLM1 | hsa-miR-145 | AC083843.1 |
| GOLM1 | hsa-miR-93 | AC083843.1 |
| GOLM1 | hsa-miR-194 | AC083843.1 |
| GPR171 | hsa-miR-32 | RP11-982M15.8 |
| GPR19 | hsa-miR-30d | MALAT1 |
| GPX1 | hsa-miR-15b | AC144450.2 |
| GRIN3A | hsa-miR-96 | MALAT1 |
| GRIN3A | hsa-miR-182 | MALAT1 |
| GSG1 | hsa-miR-192 | MALAT1 |
| GTF2IRD1 | hsa-miR-106a | RP11-661P17.1 |
| GUCY1A3 | hsa-miR-27b | AC083843.1 |
| GUCY1A3 | hsa-miR-132 | AC083843.1 |
| GUCY1A3 | hsa-miR-429 | AC083843.1 |
| GUCY2D | hsa-miR-10b | LINC00880 |
| HAS1 | hsa-miR-16 | RP11-982M15.8 |
| HDC | hsa-miR-125a-5p | RP11-982M15.8 |
| HIPK2 | hsa-miR-200a | MALAT1 |
| HMMR | hsa-miR-23a | MALAT1 |
| HMOX2 | hsa-miR-15b | AC144450.2 |
| HNRNPF | hsa-miR-199a-5p | RP11-1018N14.2 |
| HPCA | hsa-miR-106a | RP11-99H8.1 |
| HPGD | hsa-miR-33b | MALAT1 |
| IDO2 | hsa-miR-33b | AC093642.3 |
| IDUA | hsa-miR-107 | RP11-20D14.6 |
| IFNA14 | hsa-miR-143 | AC005150.1 |
| IFNA7 | hsa-miR-143 | RP11-384K6.6 |
| IL1F10 | hsa-miR-203 | RP11-982M15.8 |
| IL1RL2 | hsa-miR-107 | DRAIC |
| IL6 | hsa-miR-128 | AC083843.1 |
| IMP4 | hsa-miR-425 | DRAIC |
| ING5 | hsa-miR-218 | MALAT1 |
| INO80C | hsa-miR-214 | MALAT1 |
| INSRR | hsa-miR-181b | RP11-982M15.8 |
| IQGAP2 | hsa-miR-205 | MALAT1 |
| IRF2BP1 | hsa-miR-15b | AC144450.2 |
| IRX3 | hsa-miR-129-5p | PCGEM1 |
| ITGAE | hsa-miR-129-5p | PRCAT47 |
| ITM2A | hsa-miR-140-5p | RP11-1018N14.2 |
| JMJD4 | hsa-miR-424 | PRCAT47 |
| KATNB1 | hsa-miR-15b | AC144450.2 |
| KCNH4 | hsa-miR-15b | RP11-384K6.6 |
| KCNH8 | hsa-miR-29a | NEAT1 |
| KCNJ11 | hsa-miR-195 | LINC00937 |
| KCNJ11 | hsa-miR-193a-3p | LINC00937 |
| KIAA0101 | hsa-miR-30d | MALAT1 |
| KIF20A | hsa-miR-29a | RP11-982M15.8 |
| KIF2C | hsa-miR-195 | RP11-982M15.8 |
| KIF4B | hsa-miR-424 | AC093642.3 |
| KIF4B | hsa-miR-155 | RP11-475O6.1 |
| KIR2DL4 | hsa-miR-214 | LINC00937 |
| KLHL26 | hsa-miR-15b | AC144450.2 |
| KRT40 | hsa-miR-425 | MALAT1 |
| KRT40 | hsa-miR-181b | MALAT1 |
| KRTDAP | hsa-miR-199a-5p | PRCAT47 |
| LAMA1 | hsa-miR-30a | RP11-143A12.3 |
| LCT | hsa-miR-92a | RP11-1018N14.2 |
| LEKR1 | hsa-miR-181d | RP11-744K17.1 |
| LIG4 | hsa-miR-181b | MALAT1 |
| LINC01465 | hsa-miR-30d | DRAIC |
| LIPT1 | hsa-miR-23a | PCGEM1 |
| LOX | hsa-miR-590-5p | MALAT1 |
| LOX | hsa-miR-200b | MALAT1 |
| LOX | hsa-miR-218 | MALAT1 |
| LOX | hsa-miR-338-3p | MALAT1 |
| LSM7 | hsa-miR-15b | AC144450.2 |
| LSM7 | hsa-miR-424 | PRCAT47 |
| LY6G5C | hsa-miR-497 | RP11-982M15.8 |
| MAD2L1BP | hsa-miR-27a | CTD-3179P9.2 |
| MAD2L2 | hsa-miR-106a | AC144450.2 |
| MARCKSL1 | hsa-miR-30e | LINC01089 |
| MC1R | hsa-miR-23a | LINC01281 |
| MCCC2 | hsa-miR-181b | MALAT1 |
| MINPP1 | hsa-miR-30e | RP11-1018N14.2 |
| MKI67 | hsa-miR-125a-5p | MALAT1 |
| MKI67 | hsa-miR-222 | NEAT1 |
| MLNR | hsa-miR-15b | TINCR |
| MOG | hsa-miR-26b | AC083843.1 |
| MORN3 | hsa-miR-29b | RP11-661P17.1 |
| MPND | hsa-miR-24 | RP11-20D14.6 |
| MPZ | hsa-miR-192 | RP11-1018N14.2 |
| MRPL47 | hsa-miR-17 | PRCAT47 |
| MSC-AS1 | hsa-miR-193a-3p | LINC00937 |
| MSC-AS1 | hsa-miR-140-5p | LINC00937 |
| MSC-AS1 | hsa-miR-93 | LINC00937 |
| MUC6 | hsa-miR-125a-5p | MIR210HG |
| MYC | hsa-miR-200c | LINC01422 |
| MYC | hsa-miR-200b | RP11-475O6.1 |
| MYH7 | hsa-miR-150 | RP11-661P17.1 |
| MYT1 | hsa-miR-454 | LINC01089 |
| NAAA | hsa-miR-32 | MALAT1 |
| NAMPT | hsa-miR-106a | AC083843.1 |
| NARS | hsa-miR-15b | PRCAT47 |
| NCALD | hsa-miR-218 | MALAT1 |
| NCALD | hsa-miR-181d | MALAT1 |
| NCF2 | hsa-miR-200a | RP11-1007O24.3 |
| NETO2 | hsa-miR-199b-5p | TPTEP1 |
| NETO2 | hsa-miR-590-5p | MALAT1 |
| NETO2 | hsa-miR-150 | MALAT1 |
| NETO2 | hsa-miR-181d | MALAT1 |
| NETO2 | hsa-miR-128 | TPTEP1 |
| NFKBID | hsa-miR-27a | RP11-982M15.8 |
| NFKBIE | hsa-miR-520d-3p | AC144450.2 |
| NLRP12 | hsa-miR-106a | RP11-696F12.1 |
| NPR3 | hsa-miR-181b | MALAT1 |
| NR4A1 | hsa-miR-16 | PRCAT47 |
| NRCAM | hsa-miR-200a | MALAT1 |
| NRCAM | hsa-miR-141 | MALAT1 |
| NUSAP1 | hsa-miR-16 | NEAT1 |
| NUSAP1 | hsa-miR-15a | NEAT1 |
| OCLN | hsa-miR-18a | LINC01422 |
| OGFOD2 | hsa-miR-15b | AC144450.2 |
| OIT3 | hsa-miR-27a | RP11-1018N14.2 |
| ONECUT2 | hsa-miR-30e | NEAT1 |
| ONECUT2 | hsa-miR-29c | NEAT1 |
| ONECUT2 | hsa-miR-212 | NEAT1 |
| OR10G2 | hsa-miR-205 | RP11-3P17.4 |
| OR1I1 | hsa-miR-425 | RP11-152L7.2 |
| OR2A12 | hsa-miR-23a | RP11-1018N14.2 |
| OR2L13 | hsa-miR-92a | RP11-1018N14.2 |
| OR2T10 | hsa-miR-17 | RP11-287F9.2 |
| OR51D1 | hsa-miR-18a | RP3-340N1.2 |
| OR51E1 | hsa-miR-132 | LINC01422 |
| OR51E1 | hsa-miR-25 | AC083843.1 |
| OR51E1 | hsa-miR-200c | AC083843.1 |
| OR51E1 | hsa-miR-520d-3p | LINC01422 |
| OR51E1 | hsa-miR-143 | AC083843.1 |
| OR51E1 | hsa-miR-30c | LINC01422 |
| OR51E1 | hsa-miR-181c | MALAT1 |
| OR51E1 | hsa-miR-93 | RP11-475O6.1 |
| OR51E1 | hsa-miR-15a | LINC00937 |
| OR51G2 | hsa-miR-200a | LINC00202-1 |
| OR6C4 | hsa-miR-181c | MALAT1 |
| OR6C74 | hsa-miR-106a | RP11-287F9.2 |
| OR9A4 | hsa-miR-520d-3p | AP001476.3 |
| OS9 | hsa-miR-218 | AC144450.2 |
| P2RY10 | hsa-miR-30c | RP11-143A12.3 |
| P4HB | hsa-miR-383 | RP11-982M15.8 |
| PAGE4 | hsa-miR-23a | PRCAT47 |
| PAH | hsa-miR-23b | NEAT1 |
| PAH | hsa-miR-129-5p | NEAT1 |
| PATL2 | hsa-miR-30b | RP11-152L7.2 |
| PCDHA6 | hsa-miR-29c | LINC00937 |
| PCDHB11 | hsa-miR-32 | RP11-1018N14.2 |
| PCDHB2 | hsa-miR-153 | LINC00176 |
| PCDHB3 | hsa-miR-181b | MALAT1 |
| PCNXL3 | hsa-miR-152 | AC144450.2 |
| PCSK6 | hsa-miR-205 | RP11-475O6.1 |
| PDIA5 | hsa-miR-145 | AC083843.1 |
| PGLS | hsa-miR-15b | AC144450.2 |
| PNLIPRP1 | hsa-miR-205 | MALAT1 |
| POLR2C | hsa-miR-218 | AC144450.2 |
| PPAT | hsa-miR-24 | MALAT1 |
| PPM1H | hsa-miR-196a | NEAT1 |
| PPP1R9A | hsa-miR-206 | MALAT1 |
| PPP3CA | hsa-miR-181d | NEAT1 |
| PPP3CA | hsa-miR-429 | AC083843.1 |
| PRDX4 | hsa-miR-32 | AC093642.3 |
| PRDX4 | hsa-miR-92b | LINC01422 |
| PRF1 | hsa-miR-30d | RP11-3P17.4 |
| PRKRIR | hsa-miR-30a | MALAT1 |
| PRKRIR | hsa-miR-150 | NEAT1 |
| PRPF31 | hsa-miR-192 | RP11-1018N14.2 |
| PSG6 | hsa-miR-33b | MALAT1 |
| PTF1A | hsa-miR-92a | RP11-157E14.1 |
| PYY | hsa-miR-150 | AC002511.2 |
| RAB24 | hsa-miR-152 | AC144450.2 |
| RAB3B | hsa-miR-33b | NEAT1 |
| RAB41 | hsa-miR-30e | RP11-1018N14.2 |
| RABEP2 | hsa-miR-106a | TINCR |
| RAG2 | hsa-miR-33b | RP11-982M15.8 |
| RAPGEF5 | hsa-miR-200a | MALAT1 |
| REL | hsa-miR-203 | RP11-99H8.1 |
| REPS2 | hsa-miR-20b | NEAT1 |
| REPS2 | hsa-miR-200b | NEAT1 |
| REPS2 | hsa-miR-129-5p | NEAT1 |
| REPS2 | hsa-miR-29c | NEAT1 |
| REPS2 | hsa-miR-181b | NEAT1 |
| REPS2 | hsa-miR-96 | NEAT1 |
| REPS2 | hsa-miR-142-3p | TPTEP1 |
| REPS2 | hsa-miR-200c | NEAT1 |
| RFPL2 | hsa-miR-129-5p | DRAIC |
| RFX3 | hsa-miR-144 | NEAT1 |
| RFX6 | hsa-miR-29b | RP11-1018N14.2 |
| RMI1 | hsa-miR-128 | NEAT1 |
| RNF125 | hsa-miR-25 | MALAT1 |
| RNF148 | hsa-miR-146b-5p | RP11-982M15.8 |
| RPGRIP1 | hsa-miR-383 | MALAT1 |
| RPL18A | hsa-miR-15a | LINC00937 |
| RPL18A | hsa-miR-29b | LINC00937 |
| RPL36 | hsa-miR-24 | RP11-152L7.2 |
| RPN1 | hsa-miR-17 | RP11-287F9.2 |
| RPS27L | hsa-miR-143 | DRAIC |
| RPS6KC1 | hsa-miR-383 | MALAT1 |
| RTN4R | hsa-miR-30b | RP11-152L7.2 |
| S100A5 | hsa-miR-15b | RP11-384K6.6 |
| SAMD10 | hsa-miR-15b | AC144450.2 |
| SAT1 | hsa-miR-93 | AC144450.2 |
| SAT1 | hsa-miR-15b | TINCR |
| SCGB1D1 | hsa-miR-181a | RP11-160H22.5 |
| SCN10A | hsa-miR-128 | TINCR |
| SDK1 | hsa-miR-29a | NEAT1 |
| SEMA6B | hsa-miR-30c | RP11-152L7.2 |
| SERPINE1 | hsa-miR-205 | MALAT1 |
| SLC13A3 | hsa-miR-455-5p | MALAT1 |
| SLC16A6 | hsa-miR-129-5p | MALAT1 |
| SLC23A1 | hsa-miR-214 | RP11-3P17.4 |
| SLC35A3 | hsa-miR-206 | LINC01422 |
| SLC35A3 | hsa-miR-200c | AC083843.1 |
| SLC35F2 | hsa-miR-182 | MALAT1 |
| SLC38A11 | hsa-miR-128 | TPTEP1 |
| SLC44A5 | hsa-miR-106a | RP11-475O6.1 |
| SLC4A4 | hsa-miR-150 | NEAT1 |
| SLC9A2 | hsa-miR-205 | AC083843.1 |
| SLC9A2 | hsa-miR-145 | AC083843.1 |
| SLCO1A2 | hsa-miR-200a | MALAT1 |
| SLN | hsa-miR-194 | LINC00937 |
| SLURP1 | hsa-miR-424 | LINC01281 |
| SLURP1 | hsa-miR-16 | RP11-143A12.3 |
| SMPDL3B | hsa-miR-150 | NEAT1 |
| SMPDL3B | hsa-miR-150 | LINC01422 |
| SMS | hsa-miR-29a | NEAT1 |
| SNX11 | hsa-miR-15b | AC144450.2 |
| SOX18 | hsa-miR-7 | RP11-404O13.5 |
| SOX18 | hsa-miR-7 | LINC00880 |
| SOX18 | hsa-miR-7 | RP11-152L7.2 |
| SP5 | hsa-miR-107 | AC144450.2 |
| SPATA2L | hsa-miR-424 | RP11-982M15.8 |
| SPATC1 | hsa-miR-15b | RP11-384K6.6 |
| SPATS1 | hsa-miR-219-5p | RP11-982M15.8 |
| SPATS1 | hsa-miR-182 | MALAT1 |
| SPDEF | hsa-miR-16 | RP11-20D14.6 |
| SPDEF | hsa-miR-15b | RP11-899L11.1 |
| SPDEF | hsa-miR-497 | RP11-498P14.5 |
| SPDEF | hsa-miR-497 | RP11-982M15.8 |
| SPOCK1 | hsa-miR-135a | NEAT1 |
| SPON2 | hsa-miR-365 | LINC00937 |
| SPON2 | hsa-miR-16 | LINC00937 |
| SPRR2A | hsa-miR-133a | NEAT1 |
| SPRR2B | hsa-miR-25 | AC093642.3 |
| SPRR2B | hsa-miR-92a | RP11-71E19.1 |
| SPRR2E | hsa-miR-92a | LINC00937 |
| SS18L1 | hsa-miR-150 | RP11-661P17.1 |
| ST6GALNAC1 | hsa-miR-7 | NEAT1 |
| STAP1 | hsa-miR-23a | LINC00937 |
| STEAP4 | hsa-miR-205 | MALAT1 |
| STIL | hsa-miR-129-5p | MALAT1 |
| STIL | hsa-miR-18a | LINC01422 |
| SURF1 | hsa-miR-203 | DRAIC |
| SYT3 | hsa-miR-497 | RP11-744K17.1 |
| SYTL2 | hsa-miR-16 | RP11-3P17.4 |
| TARS | hsa-miR-150 | MALAT1 |
| TBC1D28 | hsa-miR-383 | MALAT1 |
| TBC1D30 | hsa-miR-129-5p | MALAT1 |
| TBCA | hsa-miR-212 | RP11-661P17.1 |
| TDRD1 | hsa-miR-150 | TINCR |
| TFAP2D | hsa-miR-214 | RP11-384K6.6 |
| TGDS | hsa-miR-30e | RP11-1018N14.2 |
| TGDS | hsa-miR-29c | RP11-1018N14.2 |
| THRSP | hsa-miR-106a | AP001476.3 |
| THRSP | hsa-miR-93 | RP11-99H8.1 |
| TIGIT | hsa-miR-23a | RP11-1018N14.2 |
| TIGIT | hsa-miR-30e | RP11-1018N14.2 |
| TMC5 | hsa-miR-150 | AC083843.1 |
| TMEM144 | hsa-miR-192 | MALAT1 |
| TMEM144 | hsa-miR-215 | LINC01422 |
| TMEM144 | hsa-miR-92a | LINC01422 |
| TMEM2 | hsa-miR-181d | MALAT1 |
| TNFAIP6 | hsa-miR-200a | RP11-982M15.8 |
| TOP2A | hsa-miR-96 | NEAT1 |
| TP53INP1 | hsa-miR-132 | NEAT1 |
| TP53INP1 | hsa-miR-27b | TPTEP1 |
| TP53INP1 | hsa-miR-212 | NEAT1 |
| TP53INP1 | hsa-miR-203 | TPTEP1 |
| TPX2 | hsa-miR-26a | NEAT1 |
| TRAF4 | hsa-miR-29a | MIR210HG |
| TRAT1 | hsa-miR-181b | MALAT1 |
| TRPM8 | hsa-miR-200a | NEAT1 |
| TRPM8 | hsa-miR-181d | NEAT1 |
| TRPV5 | hsa-miR-29c | RP11-661P17.1 |
| TSNARE1 | hsa-miR-15b | AC144450.2 |
| UAP1 | hsa-miR-27a | LINC00880 |
| UGT2B4 | hsa-miR-203 | TPTEP1 |
| UGT2B4 | hsa-miR-18b | LINC01422 |
| UGT2B4 | hsa-miR-203 | NEAT1 |
| UMOD | hsa-miR-223 | RP11-384K6.6 |
| UPF3A | hsa-miR-93 | RP11-99H8.1 |
| UPK1B | hsa-miR-30a | AC083843.1 |
| UPK1B | hsa-miR-200b | MALAT1 |
| VAMP8 | hsa-miR-125a-5p | AC144450.2 |
| VGLL1 | hsa-miR-29c | RP11-1018N14.2 |
| VN1R2 | hsa-miR-23b | RP11-152L7.2 |
| VPS4A | hsa-miR-195 | AC144450.2 |
| VSTM2L | hsa-miR-96 | MALAT1 |
| VWA5B1 | hsa-miR-32 | MALAT1 |
| VWA5B1 | hsa-miR-424 | MALAT1 |
| WDR5B | hsa-miR-144 | RP11-661P17.1 |
| WDR74 | hsa-miR-125a-5p | AC144450.2 |
| WNT3 | hsa-miR-146a | RP11-982M15.8 |
| XBP1 | hsa-miR-365 | LINC00176 |
| ZBED9 | hsa-miR-106b | RP11-430C7.5 |
| ZBED9 | hsa-miR-20b | AC083843.1 |
| ZBED9 | hsa-miR-20a | FLJ37035 |
| ZBED9 | hsa-miR-106b | LINC00839 |
| ZBED9 | hsa-miR-106b | LINC00937 |
| ZBTB32 | hsa-miR-15b | RP11-982M15.8 |
| ZBTB32 | hsa-miR-16 | RP11-982M15.8 |
| ZDHHC9 | hsa-miR-205 | AC083843.1 |
| ZNF133 | hsa-miR-30d | DRAIC |
| ZNF28 | hsa-miR-199a-5p | LINC00937 |
| ZNF331 | hsa-miR-24 | MALAT1 |
| ZNF331 | hsa-miR-145 | MALAT1 |
| ZNF415 | hsa-miR-33a | RP11-982M15.8 |
| ZNF443 | hsa-miR-199a-5p | LINC00937 |
| ZNF443 | hsa-miR-214 | LINC00937 |
| ZNF527 | hsa-miR-129-5p | LINC00937 |
| ZNF560 | hsa-miR-23a | RP11-982M15.8 |
| ZNF566 | hsa-miR-141 | NEAT1 |
| ZNF577 | hsa-miR-20b | NEAT1 |
| ZNF577 | hsa-miR-150 | NEAT1 |
| ZNF587 | hsa-miR-214 | LINC00937 |
| ZNF649 | hsa-miR-223 | NEAT1 |
| ZNF705G | hsa-miR-497 | NEAT1 |
| ZNF714 | hsa-miR-150 | PRCAT47 |
| ZNF716 | hsa-miR-140-5p | LINC00937 |
| ZNF750 | hsa-miR-15a | PRCAT47 |
| ZNF91 | hsa-miR-193a-3p | LINC00937 |
| ZP1 | hsa-miR-16 | RP11-982M15.8 |
| ZP3 | hsa-miR-195 | AC144450.2 |
| ZSCAN20 | hsa-miR-205 | AC083843.1 |
| ZUFSP | hsa-miR-27b | RP11-1018N14.2 |
