# Supplementary material for: The gain and loss of long noncoding RNA associated-competing endogenous RNAs in prostate cancer
Source: Oncotarget. 2016 Aug 9;7(35):57228–38. doi: 10.18632/oncotarget.11128 (PMC5302985; doi:10.18632/oncotarget.11128)
Supplement: Supplementary file 4 [file oncotarget-07-57228-s004.docx]

**Table S3. Loss ceRNA network**

| mRNA | miRNA | lncRNA |
| --- | --- | --- |
| ZZZ3 | hsa-miR-20b | MIR143HG |
| ZZZ3 | hsa-miR-106a | MIR143HG |
| ZSWIM4 | hsa-miR-93 | MIR143HG |
| ZNF786 | hsa-miR-107 | MIR143HG |
| ZNF655 | hsa-miR-19a | MEG3 |
| ZNF546 | hsa-miR-92a | MIR143HG |
| ZNF486 | hsa-miR-92a | MIR143HG |
| ZNF483 | hsa-miR-182 | RP11-475O6.1 |
| ZNF473 | hsa-miR-92a | MIR143HG |
| ZNF22 | hsa-miR-27a | AF131217.1 |
| ZNF185 | hsa-miR-206 | NEAT1 |
| ZMYND12 | hsa-miR-19a | MIR143HG |
| ZC3H12C | hsa-miR-106b | MEG3 |
| ZBTB7A | hsa-miR-93 | MIR143HG |
| ZBTB4 | hsa-miR-18b | MEG3 |
| ZBTB4 | hsa-miR-150 | MEG3 |
| XRCC6 | hsa-miR-520d-3p | MIR143HG |
| WFS1 | hsa-miR-106a | MIR99AHG |
| WFDC5 | hsa-miR-15a | AF131217.1 |
| WDR82 | hsa-miR-15a | MEG3 |
| WDR82 | hsa-miR-135a | MEG3 |
| VWA5A | hsa-miR-26b | MEG3 |
| VWA5A | hsa-miR-15b | MEG3 |
| VWA5A | hsa-miR-16 | MEG3 |
| VSTM2B | hsa-miR-19b | MIR143HG |
| VSNL1 | hsa-miR-101 | TPTEP1 |
| VPS37D | hsa-miR-34a | MIR143HG |
| VPS36 | hsa-miR-497 | MEG3 |
| VPS36 | hsa-miR-19b | MEG3 |
| VPS36 | hsa-miR-15a | MEG3 |
| VEGFA | hsa-miR-93 | MEG3 |
| VAT1 | hsa-miR-15a | MEG3 |
| VAT1 | hsa-miR-192 | MEG3 |
| UST | hsa-miR-7 | MEG3 |
| USP36 | hsa-miR-455-5p | MIR143HG |
| USP36 | hsa-miR-363 | MIR143HG |
| USP36 | hsa-miR-92a | MIR143HG |
| UQCC3 | hsa-miR-520d-3p | MIR143HG |
| UPP1 | hsa-miR-93 | MIR143HG |
| ULK4 | hsa-miR-146a | AF131217.1 |
| TSPAN2 | hsa-miR-143 | MEG3 |
| TSPAN18 | hsa-miR-15a | NEAT1 |
| TRPS1 | hsa-miR-106b | LINC01422 |
| TRIM6-TRIM34 | hsa-miR-106a | MEG3 |
| TRIM55 | hsa-miR-93 | MIR143HG |
| TRIM22 | hsa-miR-34a | MEG3 |
| TRIM2 | hsa-miR-18b | MEG3 |
| TPM2 | hsa-miR-214 | TINCR |
| TNKS | hsa-miR-192 | MEG3 |
| TNIK | hsa-miR-497 | MEG3 |
| TNIK | hsa-miR-106b | MEG3 |
| TNFRSF12A | hsa-miR-19b | MIR143HG |
| TNFRSF11A | hsa-miR-106a | MIR99AHG |
| TMEM14B | hsa-miR-133b | MEG3 |
| TMBIM1 | hsa-miR-499-5p | MEG3 |
| TFIP11 | hsa-miR-19b | MIR143HG |
| TFCP2L1 | hsa-miR-301a | MEG3 |
| TFCP2L1 | hsa-miR-130a | MEG3 |
| TFCP2L1 | hsa-miR-7 | MEG3 |
| TFCP2L1 | hsa-miR-107 | MEG3 |
| TFB2M | hsa-miR-19b | MIR143HG |
| TESK1 | hsa-miR-106a | MIR143HG |
| TESK1 | hsa-miR-93 | MIR143HG |
| TESC | hsa-miR-383 | LINC00890 |
| TCEB3 | hsa-miR-92a | MIR143HG |
| TCEB3 | hsa-miR-19b | MIR143HG |
| TCEAL4 | hsa-miR-200a | RP11-890B15.3 |
| TBL2 | hsa-miR-92a | MIR143HG |
| TBC1D21 | hsa-miR-92a | MIR143HG |
| TBC1D17 | hsa-miR-20b | MIR143HG |
| TBC1D17 | hsa-miR-20a | LINC00890 |
| SYT16 | hsa-miR-106a | MEG3 |
| SYT1 | hsa-miR-206 | MEG3 |
| SVIL | hsa-miR-375 | LINC00630 |
| STOX2 | hsa-miR-590-5p | MEG3 |
| SPTBN1 | hsa-miR-133b | MEG3 |
| SPCS1 | hsa-miR-146a | AF131217.1 |
| SOX1 | hsa-miR-27a | MIR143HG |
| SOX1 | hsa-miR-24 | MIR143HG |
| SORL1 | hsa-miR-222 | MEG3 |
| SORBS1 | hsa-miR-203 | TPTEP1 |
| SORBS1 | hsa-miR-26a | AC083843.1 |
| SLITRK6 | hsa-miR-27a | NEAT1 |
| SLITRK6 | hsa-miR-365 | NEAT1 |
| SLITRK6 | hsa-miR-19a | NEAT1 |
| SLFN12 | hsa-miR-27a | PCAT4 |
| SLC6A6 | hsa-miR-181d | MEG3 |
| SLC6A6 | hsa-miR-125a-5p | MEG3 |
| SLC6A6 | hsa-miR-133b | MEG3 |
| SLC6A6 | hsa-miR-181a | MEG3 |
| SLC5A3 | hsa-miR-499-5p | MEG3 |
| SLC2A5 | hsa-miR-499-5p | MEG3 |
| SLC14A1 | hsa-miR-19b | MEG3 |
| SLC14A1 | hsa-miR-133b | MEG3 |
| SLC14A1 | hsa-miR-145 | MEG3 |
| SESTD1 | hsa-miR-130a | MEG3 |
| SESTD1 | hsa-miR-132 | MEG3 |
| SESTD1 | hsa-miR-145 | MEG3 |
| SERPINA12 | hsa-miR-31 | AF131217.1 |
| SEMA5A | hsa-miR-125a-5p | MEG3 |
| SEMA3C | hsa-miR-23b | MEG3 |
| SEC13 | hsa-miR-146b-5p | MIR143HG |
| SDC2 | hsa-miR-200c | MEG3 |
| SCNN1A | hsa-miR-125a-5p | MEG3 |
| SCN7A | hsa-miR-23a | MEG3 |
| SAG | hsa-miR-93 | MIR143HG |
| S100A10 | hsa-miR-21 | LINC00890 |
| RHOV | hsa-miR-20a | LINC00890 |
| RGSL1 | hsa-miR-196a | MIR143HG |
| RGSL1 | hsa-miR-93 | MIR143HG |
| RGS7BP | hsa-miR-7 | MEG3 |
| RFPL3S | hsa-miR-181c | LINC00890 |
| RCOR2 | hsa-miR-106a | MIR143HG |
| RBP1 | hsa-miR-125a-5p | MEG3 |
| RBM34 | hsa-miR-106a | MIR143HG |
| RBBP6 | hsa-miR-92a | MIR143HG |
| RALY | hsa-miR-34a | MIR143HG |
| RAI14 | hsa-miR-15b | MEG3 |
| RABEP2 | hsa-miR-146b-5p | MIR143HG |
| QTRT1 | hsa-miR-7 | MIR143HG |
| PSMB6 | hsa-miR-92a | PCAT4 |
| PRRX1 | hsa-miR-106b | MEG3 |
| PRKD3 | hsa-miR-7 | MEG3 |
| PPP1R3C | hsa-miR-20a | AC083843.1 |
| PPFIBP1 | hsa-miR-23a | MEG3 |
| PPFIBP1 | hsa-miR-34a | MEG3 |
| PPFIBP1 | hsa-miR-132 | MEG3 |
| PPFIBP1 | hsa-miR-10a | MEG3 |
| PPFIBP1 | hsa-miR-19b | MEG3 |
| PPARGC1A | hsa-miR-206 | MEG3 |
| PPARGC1A | hsa-miR-222 | MEG3 |
| POLR3B | hsa-miR-92a | MIR143HG |
| POLR3B | hsa-miR-33a | MIR143HG |
| PNKP | hsa-miR-24 | LINC00890 |
| PMP22 | hsa-miR-199a-5p | TPTEP1 |
| PLN | hsa-miR-7 | NEAT1 |
| PLEKHA2 | hsa-miR-24 | MEG3 |
| PIGP | hsa-miR-520d-3p | MIR143HG |
| PIGP | hsa-miR-196b | MIR143HG |
| PIGG | hsa-miR-93 | MIR143HG |
| PHYHIPL | hsa-miR-146a | MIR143HG |
| PHYHIPL | hsa-miR-107 | MIR143HG |
| PER3 | hsa-miR-192 | MEG3 |
| PDZK1IP1 | hsa-miR-92a | PCAT4 |
| PDK4 | hsa-miR-23a | MEG3 |
| PDK3 | hsa-miR-383 | LINC00890 |
| PDE8A | hsa-miR-32 | MIR143HG |
| PDE8A | hsa-miR-93 | MIR143HG |
| PDE8A | hsa-miR-107 | MIR143HG |
| PDE8A | hsa-miR-17 | MIR143HG |
| PDE5A | hsa-miR-145 | MALAT1 |
| PCDHB12 | hsa-miR-27a | LINC00890 |
| PCDHB12 | hsa-miR-128 | LINC00890 |
| PALLD | hsa-miR-129-5p | TPTEP1 |
| PALLD | hsa-miR-23a | MEG3 |
| PAK1 | hsa-miR-15a | MEG3 |
| PAK1 | hsa-miR-27a | MEG3 |
| P3H2 | hsa-miR-93 | MEG3 |
| P2RY14 | hsa-miR-20b | MIR143HG |
| OR2T10 | hsa-miR-93 | MIR143HG |
| OPN3 | hsa-miR-455-5p | MIR143HG |
| OGDH | hsa-miR-34a | MEG3 |
| OGDH | hsa-miR-301a | MEG3 |
| ODF3B | hsa-miR-223 | LINC00890 |
| NPC2 | hsa-miR-143 | MEG3 |
| NPC2 | hsa-miR-93 | MEG3 |
| NPC2 | hsa-miR-15b | MEG3 |
| NEK9 | hsa-miR-150 | MEG3 |
| NBL1 | hsa-miR-204 | MEG3 |
| NANOG | hsa-miR-7 | MIR143HG |
| MYOT | hsa-miR-19b | MIR143HG |
| MXI1 | hsa-miR-200c | MEG3 |
| MUC21 | hsa-miR-92a | MIR143HG |
| MTERF4 | hsa-miR-93 | MIR143HG |
| MT1X | hsa-miR-93 | MIR143HG |
| MRPS16 | hsa-miR-150 | MIR143HG |
| MRPL52 | hsa-miR-520d-3p | MIR143HG |
| MRPL52 | hsa-miR-34a | MIR143HG |
| MIDN | hsa-miR-106b | LINC00890 |
| MIDN | hsa-miR-20a | LINC00890 |
| MID1 | hsa-miR-27b | TPTEP1 |
| MICALL1 | hsa-miR-31 | MIR143HG |
| MGLL | hsa-miR-29a | MIR99AHG |
| MET | hsa-miR-31 | MEG3 |
| MET | hsa-miR-130a | MEG3 |
| MED31 | hsa-miR-129-5p | MIR99AHG |
| MCAM | hsa-miR-93 | NEAT1 |
| MBTPS1 | hsa-miR-22 | MEG3 |
| MARK4 | hsa-miR-20b | MIR143HG |
| MARK2 | hsa-miR-34a | MIR143HG |
| MAPRE1 | hsa-miR-301a | MEG3 |
| MAP1S | hsa-miR-128 | MIR143HG |
| LRRC74B | hsa-miR-520d-3p | MIR143HG |
| LRRC20 | hsa-miR-106a | MIR143HG |
| LRRC20 | hsa-miR-32 | MIR143HG |
| LRRC20 | hsa-miR-17 | MIR143HG |
| LRRC20 | hsa-miR-92a | MIR143HG |
| LPAR1 | hsa-miR-26b | RP11-475O6.1 |
| LIMA1 | hsa-miR-23a | MEG3 |
| LIMA1 | hsa-miR-93 | MEG3 |
| LIMA1 | hsa-miR-455-5p | MEG3 |
| LIMA1 | hsa-miR-125a-5p | MEG3 |
| LHX6 | hsa-miR-20b | MIR143HG |
| LECT2 | hsa-miR-20b | MIR143HG |
| LAX1 | hsa-miR-192 | MIR143HG |
| KRT75 | hsa-miR-16 | EPB41L4A-AS1 |
| KRT75 | hsa-miR-195 | EPB41L4A-AS1 |
| KRT75 | hsa-miR-23b | EPB41L4A-AS1 |
| KRT15 | hsa-miR-204 | MEG3 |
| KLHL5 | hsa-miR-19b | MEG3 |
| KLF2 | hsa-miR-92a | MIR143HG |
| KIAA0430 | hsa-miR-135a | MEG3 |
| JPH2 | hsa-miR-24 | MEG3 |
| JOSD2 | hsa-miR-34a | MIR143HG |
| ITGB6 | hsa-miR-145 | MEG3 |
| ITGB6 | hsa-miR-98 | MEG3 |
| ITGB6 | hsa-miR-22 | MEG3 |
| ITGB4 | hsa-miR-15a | MEG3 |
| ITGA5 | hsa-miR-22 | NEAT1 |
| ITGA2 | hsa-miR-10a | MEG3 |
| INPP5F | hsa-miR-150 | MEG3 |
| INPP1 | hsa-miR-16 | AF131217.1 |
| IMPA2 | hsa-miR-25 | MIR143HG |
| IGFBP4 | hsa-miR-129-5p | MIR99AHG |
| IGFBP4 | hsa-miR-93 | MIR99AHG |
| IER3 | hsa-miR-20b | MIR143HG |
| IDS | hsa-miR-15b | MEG3 |
| IDI2 | hsa-miR-520d-3p | MIR143HG |
| HSPBP1 | hsa-miR-7 | MIR143HG |
| HNRNPF | hsa-miR-19b | MIR143HG |
| HMGCS1 | hsa-miR-223 | MEG3 |
| HMGCS1 | hsa-miR-19b | MEG3 |
| HMG20A | hsa-miR-107 | MEG3 |
| HIST2H2BE | hsa-miR-92a | MIR143HG |
| HES6 | hsa-miR-34a | MIR143HG |
| HEG1 | hsa-miR-24 | MEG3 |
| HDGFRP3 | hsa-miR-107 | MEG3 |
| HAND1 | hsa-miR-363 | MIR143HG |
| HADHB | hsa-miR-33b | MIR143HG |
| GSTM4 | hsa-miR-125a-5p | MEG3 |
| GSTM3 | hsa-miR-193a-3p | RP11-475O6.1 |
| GSTM2 | hsa-miR-150 | MEG3 |
| GSTM1 | hsa-miR-204 | MEG3 |
| GPX1 | hsa-miR-195 | PCAT4 |
| GPS2 | hsa-miR-31 | MIR99AHG |
| GPR183 | hsa-miR-218 | LINC00890 |
| GPR161 | hsa-miR-29a | MIR99AHG |
| GOLGA8B | hsa-miR-19a | MEG3 |
| GNPTAB | hsa-miR-19b | MEG3 |
| GNLY | hsa-miR-24 | LINC00890 |
| GNAI1 | hsa-miR-203 | LINC00890 |
| GLTP | hsa-miR-7 | MEG3 |
| GLT8D2 | hsa-miR-338-3p | RP11-890B15.3 |
| GLT8D2 | hsa-miR-182 | RP11-890B15.3 |
| GLI3 | hsa-miR-143 | MEG3 |
| GJA1 | hsa-miR-93 | TPTEP1 |
| GJA1 | hsa-miR-219-5p | TPTEP1 |
| GIPC1 | hsa-miR-33a | MIR143HG |
| GIMAP7 | hsa-miR-27b | PCAT4 |
| GGA2 | hsa-miR-499-5p | MEG3 |
| GAD1 | hsa-miR-33a | MIR143HG |
| GAD1 | hsa-miR-92a | MIR143HG |
| GAD1 | hsa-miR-192 | MIR143HG |
| GABRP | hsa-miR-98 | MEG3 |
| GABRP | hsa-miR-10b | MEG3 |
| GABRP | hsa-miR-24 | MEG3 |
| FZD3 | hsa-miR-106a | MEG3 |
| FRMPD2 | hsa-miR-19b | MIR143HG |
| FOXP2 | hsa-miR-206 | MEG3 |
| FOXA3 | hsa-miR-107 | LINC00890 |
| FOXA3 | hsa-miR-27a | LINC00890 |
| FOXA3 | hsa-miR-27b | LINC00890 |
| FLNC | hsa-miR-125a-5p | RP11-88H9.2 |
| FIBIN | hsa-miR-18b | MEG3 |
| FGFR2 | hsa-miR-106a | NEAT1 |
| FGF4 | hsa-miR-33a | MIR143HG |
| FBXO32 | hsa-miR-19a | MEG3 |
| FBXO15 | hsa-miR-92b | MIR143HG |
| FAT2 | hsa-miR-7 | MEG3 |
| FAM71F1 | hsa-miR-93 | MIR143HG |
| FAM50B | hsa-miR-93 | MIR143HG |
| FAM169A | hsa-miR-150 | MEG3 |
| FAM168A | hsa-miR-150 | MIR143HG |
| FA2H | hsa-miR-19b | MIR143HG |
| FA2H | hsa-miR-93 | MIR143HG |
| EYA4 | hsa-miR-19b | MEG3 |
| EVPLL | hsa-miR-93 | MIR143HG |
| EPS8 | hsa-miR-23a | MEG3 |
| EPS8 | hsa-miR-10a | MEG3 |
| EPHB4 | hsa-miR-499-5p | MEG3 |
| EPHA2 | hsa-miR-98 | MIR143HG |
| ENAH | hsa-miR-23a | MEG3 |
| EIF4EBP2 | hsa-miR-15a | MEG3 |
| EIF2AK1 | hsa-miR-19b | MIR143HG |
| EFNA5 | hsa-miR-128 | MEG3 |
| EDNRA | hsa-miR-200c | MALAT1 |
| DUOXA1 | hsa-miR-145 | MEG3 |
| DUOX1 | hsa-miR-140-5p | MEG3 |
| DRG2 | hsa-miR-24 | MIR143HG |
| DOK4 | hsa-miR-125a-5p | MEG3 |
| DNAJB5 | hsa-miR-23a | MEG3 |
| DHX8 | hsa-miR-195 | PCAT4 |
| DCK | hsa-miR-92a | MIR143HG |
| DCBLD2 | hsa-miR-181c | MEG3 |
| DCBLD2 | hsa-miR-7 | MEG3 |
| CYP4B1 | hsa-miR-218 | MEG3 |
| CYP3A5 | hsa-miR-125a-5p | MEG3 |
| CYBRD1 | hsa-miR-143 | MEG3 |
| CXXC1 | hsa-miR-24 | MIR143HG |
| CXCL14 | hsa-miR-106a | MIR143HG |
| CRISPLD2 | hsa-miR-15a | MEG3 |
| CRISPLD2 | hsa-miR-143 | MEG3 |
| CRISPLD2 | hsa-miR-214 | RP11-475O6.1 |
| CRIPT | hsa-miR-24 | LINC00890 |
| CRIM1 | hsa-miR-27a | MEG3 |
| CPT1A | hsa-miR-499-5p | MEG3 |
| CPA6 | hsa-miR-27a | MEG3 |
| CORO2B | hsa-miR-146a | MIR143HG |
| COMMD6 | hsa-miR-20a | LINC00890 |
| CNTN1 | hsa-miR-29a | AC083843.1 |
| CNN1 | hsa-miR-15a | RP11-475O6.1 |
| CKB | hsa-miR-15a | RP11-475O6.1 |
| CKB | hsa-miR-16 | LINC00630 |
| CILP | hsa-miR-106a | MIR143HG |
| CERCAM | hsa-miR-140-5p | RP11-890B15.3 |
| CDCA5 | hsa-miR-19b | MIR143HG |
| CDC42EP3 | hsa-miR-301a | TPTEP1 |
| CD34 | hsa-miR-24 | MIR143HG |
| CD28 | hsa-miR-93 | MIR143HG |
| CCNI2 | hsa-miR-27a | MEG3 |
| CCND2 | hsa-miR-424 | TPTEP1 |
| CCL13 | hsa-miR-33a | MIR143HG |
| CAV1 | hsa-miR-200b | NEAT1 |
| CAV1 | hsa-miR-203 | NEAT1 |
| CAV1 | hsa-miR-199b-5p | NEAT1 |
| CAV1 | hsa-miR-106a | TPTEP1 |
| CASP8 | hsa-miR-20b | MIR143HG |
| CASP2 | hsa-miR-107 | MIR143HG |
| CAP2 | hsa-miR-7 | MEG3 |
| CAMK2D | hsa-miR-106b | MEG3 |
| CALD1 | hsa-miR-20a | AC083843.1 |
| C9orf62 | hsa-miR-34a | MIR143HG |
| C8orf37 | hsa-miR-98 | MIR143HG |
| C14orf93 | hsa-miR-27b | LINC00890 |
| BPNT1 | hsa-miR-20b | MIR143HG |
| BPI | hsa-miR-520d-3p | MIR143HG |
| BPHL | hsa-miR-92a | MIR143HG |
| BPGM | hsa-miR-106a | LINC00890 |
| BNC2 | hsa-miR-7 | MEG3 |
| BLK | hsa-miR-92a | MIR143HG |
| BCL2 | hsa-miR-200c | MEG3 |
| BAD | hsa-miR-34a | RP11-400K9.4 |
| AZGP1 | hsa-miR-107 | MIR143HG |
| AZGP1 | hsa-miR-129-5p | MIR99AHG |
| AVPI1 | hsa-miR-93 | MIR143HG |
| ASL | hsa-miR-19b | MIR143HG |
| ARSJ | hsa-miR-34a | MEG3 |
| ARSJ | hsa-miR-145 | MEG3 |
| ARSJ | hsa-miR-26a | MEG3 |
| ARHGAP20 | hsa-miR-23a | MEG3 |
| AQP3 | hsa-miR-204 | MEG3 |
| APOBEC3H | hsa-miR-520d-3p | MIR143HG |
| APOA5 | hsa-miR-93 | MIR143HG |
| APCS | hsa-miR-106a | MIR143HG |
| AP1M2 | hsa-miR-150 | LINC00890 |
| ANXA6 | hsa-miR-129-5p | MEG3 |
| ANO6 | hsa-miR-33a | NEAT1 |
| ANO6 | hsa-miR-200c | NEAT1 |
| ALDH1A1 | hsa-miR-429 | MEG3 |
| AKAP3 | hsa-miR-199a-5p | PCAT4 |
| ADCY2 | hsa-miR-125a-5p | MEG3 |
| ADAMTS17 | hsa-miR-33a | MIR143HG |
| ACSS3 | hsa-miR-129-5p | AC083843.1 |
| ACSS3 | hsa-miR-26a | AC083843.1 |
| ACSS3 | hsa-miR-24 | MEG3 |
| ACSS3 | hsa-miR-143 | MEG3 |
| ACSF2 | hsa-miR-204 | MEG3 |
| ABTB1 | hsa-miR-93 | MIR143HG |
| ABLIM2 | hsa-miR-92a | MIR143HG |
| ABHD14B | hsa-miR-106a | MIR143HG |
| ABHD12 | hsa-miR-107 | MIR143HG |
| ABCC9 | hsa-miR-18b | MEG3 |
| AADACL3 | hsa-miR-93 | MIR143HG |
