# Supplementary material for: The gain and loss of long noncoding RNA associated-competing endogenous RNAs in prostate cancer
Source: Oncotarget. 2016 Aug 9;7(35):57228–38. doi: 10.18632/oncotarget.11128 (PMC5302985; doi:10.18632/oncotarget.11128)
Supplement: Supplementary file 5 [file oncotarget-07-57228-s005.docx]

**Table S5. ceRNA pairs significantly correlatied with overall survival of PC patients in gain network**

| Log Rank P value | mRNA | miRNA | lncRNA |
| --- | --- | --- | --- |
| 2.23E-05 | ADAMTS3 | hsa-miR-144 | MALAT1 [[1](#_ENREF_1)] |
| 0.00086 | VWA5B1 | hsa-miR-32 | MALAT1 |
| 0.00358 | EEF1A2 [[2](#_ENREF_2)] | hsa-miR-31 | RP11-384K6.6 |
| 0.00396 | CA2 | hsa-miR-106a | LINC00937 |
| 0.00772 | MKI67 [[3](#_ENREF_3)] | hsa-miR-125a-5p | MALAT1 |
| 0.00828 | PAH | hsa-miR-23b | NEAT1 |
| 0.00971 | GPR19 [[4](#_ENREF_4)] | hsa-miR-30d | MALAT1 |
| 0.0113 | P4HB [[5](#_ENREF_5), [6](#_ENREF_6)] | hsa-miR-383 | RP11-982M15.8 |
| 0.0136 | CNTNAP2 | hsa-miR-200b | MALAT1 |
| 0.0143 | EEF1A2 [[2](#_ENREF_2)] | hsa-miR-31 | RP11-384K6.6 |
| 0.0164 | RNF148 | hsa-miR-146b-5p | RP11-982M15.8 |
| 0.0173 | CIDEC | hsa-miR-29b | RP11-982M15.8 |
| 0.0189 | KIF20A | hsa-miR-29a | RP11-982M15.8 |
| 0.022 | FOXA1 [[7](#_ENREF_7)] | hsa-miR-93 | AC091814.3 |
| 0.0225 | IL6 [[8](#_ENREF_8)] | hsa-miR-128 | AC083843.1 |
| 0.0239 | EGR2 [[9](#_ENREF_9)] | hsa-miR-93 | MALAT1 |
| 0.0241 | SPDEF [[10](#_ENREF_10)] | hsa-miR-497 | RP11-982M15.8 |
| 0.0313 | ZP1 | hsa-miR-16 | RP11-982M15.8 |
| 0.0313 | IL1F10 | hsa-miR-203 | RP11-982M15.8 |
| 0.0313 | SPATA2L | hsa-miR-424 | RP11-982M15.8 |
| 0.032 | LY6G5C | hsa-miR-497 | RP11-982M15.8 |
| 0.032 | RNF148 | hsa-miR-146b-5p | RP11-982M15.8 |
| 0.032 | INSRR | hsa-miR-181b | RP11-982M15.8 |
| 0.032 | CYP7A1 [[11](#_ENREF_11)] | hsa-miR-181d | RP11-982M15.8 |
| 0.032 | NFKBID | hsa-miR-27a | RP11-982M15.8 |
| 0.032 | KIF2C | hsa-miR-195 | RP11-982M15.8 |
| 0.032 | ZNF560 | hsa-miR-23a | RP11-982M15.8 |
| 0.0327 | B9D1 | hsa-miR-10b | RP11-982M15.8 |
| 0.0343 | ADAMTS3 | hsa-miR-101 | MALAT1 |
| 0.0357 | INSRR | hsa-miR-181b | RP11-982M15.8 |
| 0.0357 | F7 | hsa-miR-10a | RP11-982M15.8 |
| 0.0364 | B9D1 | hsa-miR-10b | RP11-982M15.8 |
| 0.0371 | NFKBID | hsa-miR-27a | RP11-982M15.8 |
| 0.0423 | C1QL4 | hsa-miR-10a | RP11-982M15.8 |
| 0.0427 | B9D1 | hsa-miR-10b | RP11-982M15.8 |
| 0.0467 | SPDEF | hsa-miR-497 | RP11-982M15.8 |
| 0.0482 | AP1M2 | hsa-miR-150 | PRCAT47 |
| 0.0482 | PAGE4 [[12](#_ENREF_12)] | hsa-miR-23a | PRCAT47 |
| 0.0482 | MRPL47 | hsa-miR-17 | PRCAT47 |
| 0.0496 | DEFB121 | hsa-miR-7 | CASC17 |
